# MiR-4646-5p Acts as a Tumor-Suppressive Factor in Triple Negative Breast Cancer and Targets the Cholesterol Transport Protein GRAMD1B

**DOI:** 10.3390/ncrna10010002

**Published:** 2023-12-26

**Authors:** Katharina Jonas, Felix Prinz, Manuela Ferracin, Katarina Krajina, Alexander Deutsch, Tobias Madl, Beate Rinner, Ondrej Slaby, Christiane Klec, Martin Pichler

**Affiliations:** 1 Division of Oncology, Department of Internal Medicine, Medical University of Graz, 8036 Graz, Austria; katharina.jonas@medunigraz.at (K.J.);; 2 Research Unit for Non-Coding RNA and Genome Editing, Medical University of Graz, 8010 Graz, Austria; 3 Department of Medical and Surgical Sciences (DIMEC), University of Bologna, 40126 Bologna, Italy; 4 Translational Oncology, II. Med Clinics Hematology and Oncology, 86156 Augsburg, Germany; 5 Division of Hematology, Department of Internal Medicine, Medical University of Graz, 8036 Graz, Austria; 6 Division of Molecular Biology & Biochemistry, Gottfried Schatz Research Center for Cell Signaling, Metabolism and Aging, Medical University of Graz, 8010 Graz, Austria; 7 BioTechMed-Graz, 8010 Graz, Austria; 8 Department for Biomedical Research, Medical University of Graz, 8036 Graz, Austria; 9 Department of Biology, Faculty of Medicine and Central European Institute of Technology, Masaryk University, 625 00 Brno, Czech Republic

**Keywords:** microRNA (miRNA), triple negative breast cancer (TNBC), cholesterol transport protein, GRAM domain-containing protein 1B (GRAMD1B)

## Abstract

MicroRNAs (miRNAs) are crucial post-transcriptional regulators of gene expression, and their deregulation contributes to many aspects of cancer development and progression. Thus, miRNAs provide insight into oncogenic mechanisms and represent promising targets for new therapeutic approaches. A type of cancer that is still in urgent need of improved treatment options is triple negative breast cancer (TNBC). Therefore, we aimed to characterize a novel miRNA with a potential role in TNBC. Based on a previous study, we selected miR-4646-5p, a miRNA with a still unknown function in breast cancer. We discovered that higher expression of miR-4646-5p in TNBC patients is associated with better survival. In vitro assays showed that miR-4646-5p overexpression reduces growth, proliferation, and migration of TNBC cell lines, whereas inhibition had the opposite effect. Furthermore, we found that miR-4646-5p inhibits the tube formation ability of endothelial cells, which may indicate anti-angiogenic properties. By whole transcriptome analysis, we not only observed that miR-4646-5p downregulates many oncogenic factors, like tumor-promoting cytokines and migration- and invasion-related genes, but were also able to identify a direct target, the GRAM domain-containing protein 1B (GRAMD1B). GRAMD1B is involved in cellular cholesterol transport and its knockdown phenocopied the growth-reducing effects of miR-4646-5p. We thus conclude that GRAMD1B may partly contribute to the diverse tumor-suppressive effects of miR-4646-5p in TNBC.

## 1. Introduction

According to the World Health Organization, breast cancer is the most common type of cancer among women worldwide and the leading cause of cancer-related deaths in women. Triple negative breast cancer (TNBC), which accounts for around 12–17% of all breast cancer cases, is a subtype that stands out as particularly aggressive and difficult to treat due to the absence of the classic therapeutic targets estrogen receptor (ER), progesterone receptor (PR), and human epidermal growth factor receptor-2 (HER2) [1,2]. This highlights the urgent need to further elucidate mechanisms of TNBC carcinogenesis to eventually enable the development of new therapeutic approaches.

With this in mind, one may explore the world of non-coding RNAs (ncRNAs), as it is well established by now that the vast majority of the genome does not harbor protein-coding genes but is transcribed into various types of ncRNAs that execute crucial regulatory functions [3,4,5]. One class of highly conserved ncRNAs encompasses microRNAs (miRNAs), which can be distinguished by their short length of around 18 to 22 nucleotides [4,6,7]. MiRNAs are transcribed as long mono- or polycistronic pri-miRNAs, which are subsequently cleaved by the Drosha-DGCR8 complex into stem–loop precursor miRNAs (pre-miRNAs) [8,9,10]. These pre-miRNAs are exported from the nucleus and trimmed by a second RNase III endonuclease called Dicer into short miRNA duplexes [8,10,11]. One strand of these duplexes is finally loaded into the RNA-induced silencing complex (RISC) and, by binding to mRNA targets via a short seed region, mostly within their 3′ untranslated region (UTR), induces degradation or translational repression of the mRNA [12,13]. In addition to the classical biogenesis pathway of miRNAs, there is also a Drosha-independent route where spliced and debranched introns that resemble the pre-miRNA structure are directly processed by Dicer into miRNA duplexes while bypassing the cleavage step by Drosha [14]. These miRNAs are called mirtronic miRNAs [14].

Since their discovery, miRNAs have received a lot of attention in research due to their involvement in development and diseases. Notably, miRNAs are frequently deregulated in cancer and can contribute to almost all hallmarks of cancer [15,16,17,18]. To name but a few examples, miRNAs have been found to regulate proliferation, migration, invasion, metastasis, and stemness of breast cancer cells either in a tumor-suppressive or oncogenic fashion [19,20,21,22,23]. Moreover, they were found to have the potential to serve as clinical biomarkers and therapeutic targets [24,25,26,27].

We thus aimed to functionally characterize the role of a novel miRNA in TNBC named miR-4646-5p. We selected this particular miRNA based on the fact that its expression in breast cancer had already been previously described using mammospheres, three-dimensional tumorspheres that were generated from breast cancer cell lines [19]. This mammosphere model is of interest because it facilitates the enrichment of breast cancer cells with a stem cell-like character, whose percentage is also increased in TNBC compared to other breast cancer subtypes, which contributes to the aggressiveness of TNBC [28,29]. MiR-4646-5p has recently been described as a mirtronic miRNA, as it originates from a spliced intron of its host gene abhydrolase domain-containing 16A (*ABHD16A*) and skips Drosha cleavage [30]. While miR-4646-5p has been associated with gastric cancer metastasis [30], its function in breast cancer has not been explored until now.

In the present study, we found that miR-4646-5p affects the growth, proliferation, apoptosis, and migration of TNBC cell lines. Moreover, the miRNA also exerted an effect on the in vitro tube formation of endothelial cells, hinting at a role in tumor angiogenesis. The observed cellular phenotypes may, in part, be explained by a target of miR-4646-5p that we identified, the GRAM domain-containing protein 1B (GRAMD1B), which is involved in cellular cholesterol transport. Moreover, broad effects on cytokine signaling may also contribute to the cellular effects of this miRNA.

## 2. Results

### 2.1. MiR-4646-5p Levels Are Associated with Survival in TNBC Patients, and Ectopic miR-4646-5p Overexpression Reduces Growth and Proliferation, and Induces Apoptosis in TNBC Cells

**Figure 1:**
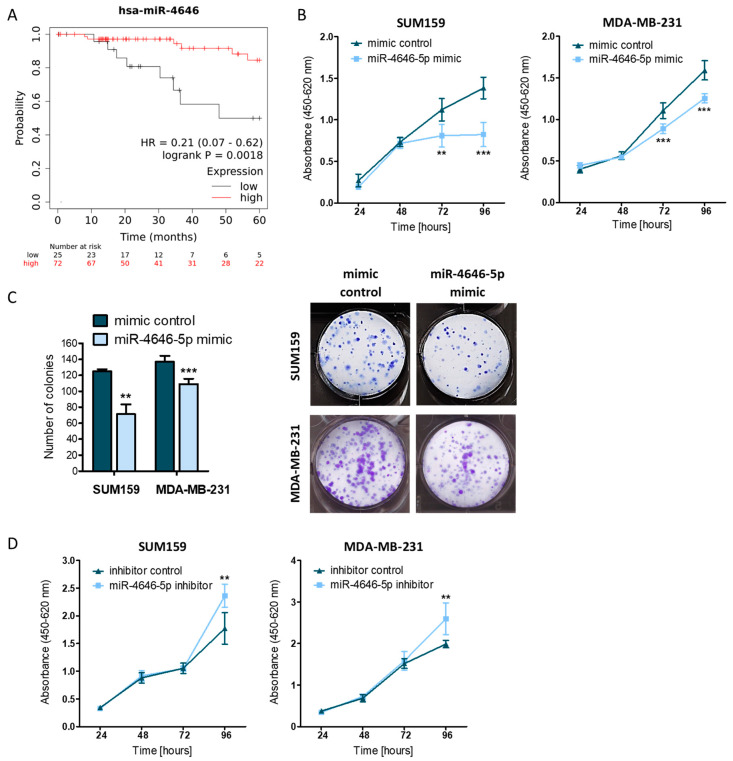
MiR-4646-5p is associated with better survival of triple negative breast cancer (TNBC) patients and reduces growth of TNBC cell lines. (**A**) Overall survival of patients with TNBC split into a miR-4646 low- (black; *n* = 25) and high-expression (red; *n* = 72) group (log-rank test; *p* = 0.0018; HR—Hazard Ratio). (**B**) The impact of transient miR-4646-5p mimic transfection on cell growth was assessed by WST-1 assays in two TNBC cell lines (*n* = 6; mean ± SD; ** *p* ≤ 0.01, *** *p* ≤ 0.001). (**C**) SUM159 and MDA-MB-231 cells transiently transfected with miR-4646-5p mimic or control were seeded at low density, and colony formation was observed after 7 and 14 days, respectively. Absolute numbers of colonies (representative images on the right) were counted and are depicted as mean ± SD (*n* = 3 for SUM159, *n* = 6 for MDA-MB-231; ** *p* ≤ 0.01; *** *p* ≤ 0.001). (**D**) The effect of transient miR-4646-5p inhibitor transfection on cell growth was assessed in WST-1 assays (*n* = 6; mean ± SD; ** *p* ≤ 0.01).

In order to clarify whether miR-4646-5p plays a potential role in breast cancer, we first assessed its clinical relevance in patients. To this end, we analyzed data from a triple negative breast cancer (TNBC) patient cohort from the Cancer Genome Atlas Project (TCGA) using a publicly available survival analysis tool [31]. Higher expression levels of the *miR-4646* gene were associated with significantly longer overall survival (log-rank test; *p* = 0.0018; Hazard ratio = 0.21; 95% confidence interval 0.07–0.62) of TNBC patients (Figure 1A). In addition, higher expression of the *miR-4646* host gene *ABHD16A* was connected to a (non-significant) trend for better overall survival of patients with TNBC and a significant difference in patients with basal breast cancer (Supplementary Figure S1A,B).

Based on this finding, we started an in vitro characterization of the biological role of miR-4646-5p in TNBC cells by gain- and loss-of-function experiments using a synthetic miR-4646-5p mimic and a miR-4646-5p inhibitor, respectively. RT-qPCR was used to confirm overexpression/knockdown efficiency. Melting curve analysis showed specific amplification of overlapping PCR products of both the mimic as well as the endogenous miRNA by the employed primers, attesting to its specificity (Supplementary Figure S2A). While the mimic transfection led to a strong upregulation of miR-4646-5p in the two TNBC cell lines SUM159 and MDA-MB-231, the inhibitor transfection did not result in a decrease detectable by RT-qPCR (Supplementary Figure S2B,C). However, binding of the locked nucleic acid (LNA) inhibitor to the miRNA does not necessarily induce degradation of the miRNA but primarily functions by sequestering the miRNA and blocking the interaction with target mRNAs, meaning the miRNA can still be detected by RT-qPCR at unchanged levels despite its functional inhibition [32]. To prove this consideration, once we had identified a binding target of miR-4646-5p, we confirmed the functionality of the inhibitor in a luciferase reporter assay (Supplementary Figure S2E).

As high levels of *miR-4646* were associated with better patient outcomes, we started with gain-of-function experiments. Cellular growth assays over 96 h showed that the transient upregulation of miR-4646-5p by mimic transfection resulted in decreased growth of two TNBC cell lines 72 and 96 h after the transfection (Figure 1B). We confirmed the impact on cellular growth over a longer period by colony formation assays, where cells were cultured for 7 to 14 days (depending on the cell line), and observed reduced numbers of SUM159 and MDA-MB-231 colonies upon miR-4646-5p mimic transfection (Figure 1C). Finally, to complement our gain of function findings, we applied a miR-4646-5p inhibitor and could observe increased growth after 96 h, the opposite effect of miR-4646-5p overexpression (Figure 1D).

**Figure 2:**
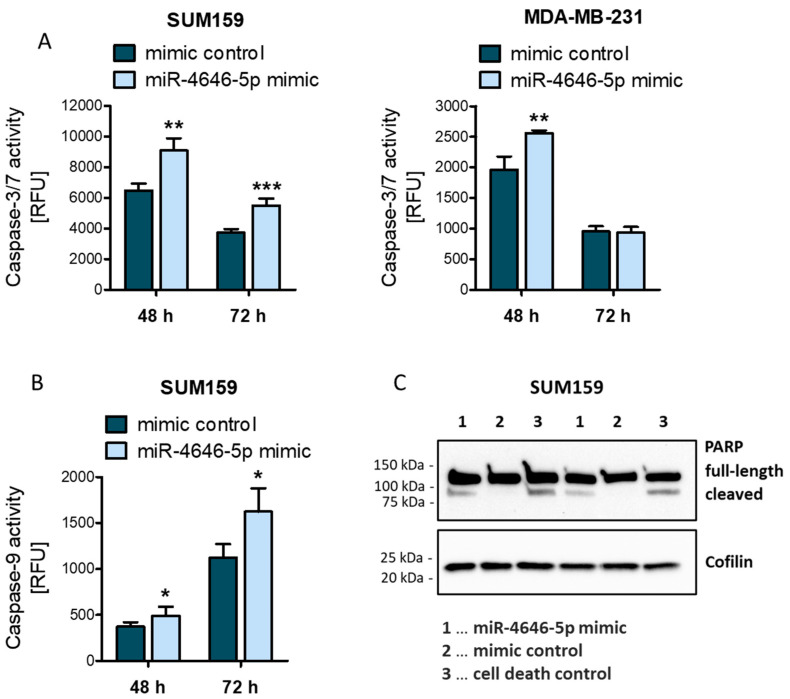
MiR-4646-5p can induce apoptosis in a cell line-specific context. (**A**) Luminescent caspase-3/7 activity assays were performed 48 h and 72 h after miR-4646-5p mimic or control transfection to investigate apoptosis induction in SUM159 and MDA-MB-231 cells (*n* = 4 for 48 h; *n* = 6 for 72 h; mean ± SD; ** *p* ≤ 0.01, *** *p* ≤ 0.001; RFU = relative fluorescence unit). (**B**) Luminescent caspase-9 activity assays were performed 48 h and 72 h after miR-4646-5p mimic or control transfection to investigate apoptosis induction in SUM159 cells (*n* = 6; mean ± SD; * *p* ≤ 0.05 RFU = relative fluorescence unit). (**C**) The cleavage of PARP, a process indicative of apoptosis, was detected by Western blotting 48 h after transient miR-4646-5p mimic, negative mimic control, or positive cell death control transfection of SUM159 cells.

To identify the mode of action behind the change in cellular growth, we performed EdU proliferation assays and detected a consistent reduction in proliferation induced by the miR-4646-5p mimic in both cell lines (Supplementary Figure S3). We also investigated the possibility of apoptosis induction as an additional mechanism causing reduced cell numbers. To this end, we performed caspase-3/7 and caspase-9 activity assays. Mimic transfection increased the activity of the two effector caspases-3 and -7 significantly in SUM159 cells 48 and 72 h after transfection, whereas in MDA-MB-231 cells, an increase was seen only after 48 h (Figure 2A). Similarly, caspase-9 showed increased activity in SUM159 cells after 48 and 72 h (Figure 2B) and the induction of apoptosis could furthermore be confirmed by Western blotting for cleaved PARP, a substrate of the effector caspase-7 (Figure 2C). Of note, for the MDA-MB-231 cell line, we could not detect differences in caspase-9 activity or increased signs of PARP cleavage upon miR-4646-5p overexpression (Supplementary Figure S4A,B). It thus seems that the impact of miR-4646-5p on apoptosis is cancer cell line-specific, which might be caused by genetic differences and differences in the expression of direct and indirect targets of the miRNA in the TNBC cells. We further investigated this by screening the expression of several pro-apoptotic genes by RT-qPCR, namely of the two pro-apoptotic effectors *BAX* and *BAK*, the BH3-only activator *PUMA*, and the two BH3-only sensitizers *BAD* and *NOXA*. We could not detect expression of *BAK* and *NOXA* in SUM159 cells but the miR-4646-5p mimic induced a significant upregulation of *BAX* and *BAD* (Supplementary Figure S4C). MDA-MB-231 cells did express *BAK* and *NOXA* but did not exhibit any changes in the expression of these pro-apoptotic genes in response to miR-4646-5p overexpression (Supplementary Figure S4C). This supports the notion that the two cell lines exhibit inherent differences that may also be responsible for disparities in the impact of miR-4646-5p on apoptosis. We concluded that the mechanisms behind the reduced cellular growth patterns caused by miR-4646-5p may be different in different cellular contexts, a phenomenon not unheard of for miRNAs [33], and may additionally be influenced by other effects (proliferation, autophagy, senescence, etc.).

### 2.2. MiR-4646-5p Reduces the Migration of TNBC Cells

**Figure 3:**
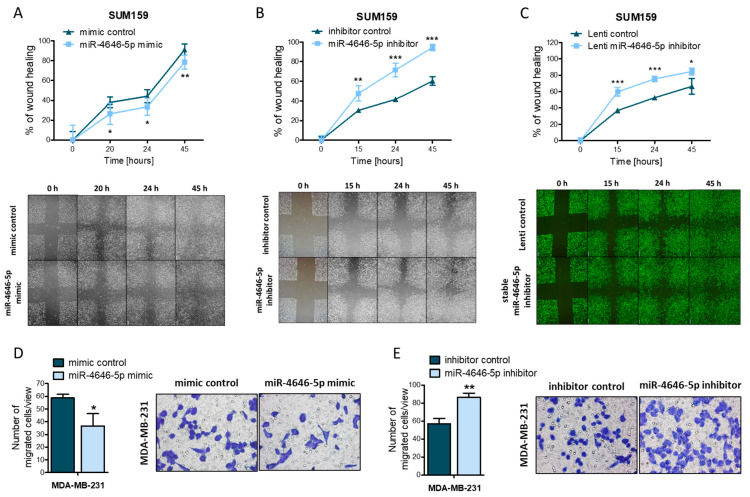
MiR-4646-5p reduces migration of TNBC cell lines. The effect of (**A**) transient miR-4646-5p mimic, (**B**) transient inhibitor transfection, or (**C**) stable miR-4646-5p inhibition on the migration of SUM159 cells was investigated in scratch assays (representative images at the bottom). For each time point, remaining areas relative to the 0 h time point were calculated and are depicted as mean ± SD (*n* = 4; (**A**) *n* = 8; * *p* ≤ 0.05, ** *p* ≤ 0.01, *** *p* ≤ 0.001). Transwell migration assays with MDA-MB-231 cells were performed to confirm the impact of (**D**) the miR-4646-5p mimic or (**E**) miR-4646-5p inhibitor on cell migration. In each transwell, cells were counted in five representative fields of view (representative images on the right) and are shown as mean ± SD (*n* = 3; * *p* ≤ 0.05, ** *p* ≤ 0.01).

Aside from growth and proliferation, cell migration is an important aspect of cancer cell biology, as it constitutes a fundamental requirement for the spread, dissemination, and eventual metastasis formation of tumors [34]. Therefore, we investigated the effect of miR-4646-5p on the in vitro migration of SUM159 cells using a wound healing assay (the MDA-MB-231 cell line was not suitable for this analysis, as cells detached easily and re-seeded in the scratched area, thereby masking actual migratory effects). Mimic overexpression of miR-4646-5p significantly delayed the spreading of the cells and the closure of the introduced scratch (Figure 3A). Conversely, transient inhibition of miR-4646-5p favored wound closure (Figure 3B). In order to corroborate our findings of transient mimic and inhibitor transfections, we generated a stable miR-4646-5p inhibitor expressing SUM159 cell line by lentiviral transfection (Supplementary Figure S2D) (a stable miR-4646-5p overexpression cell line could not be generated due to the cell death-inducing effect of miR-4646-5p overexpression). With these stable miR-4646-5p inhibitor cells, we could reproduce the effect of increased migration (Figure 3C).

To further confirm our findings by an independent experimental approach, we employed transwell migration assays. Also, in this setting, we observed that miR-4646-5p upregulation led to a significant reduction in the number of migrated MDA-MB-231 cells and a (non-significant) reduction in migrated SUM159 cells (Figure 3D, Supplementary Figure S5A), while miR-4646-5p inhibition significantly increased cell migration through the permeable membrane (Figure 3E, Supplementary Figure S5B).

### 2.3. MiR-4646-5p Reduces In Vitro Tube Formation of Endothelial Cells

**Figure 4:**
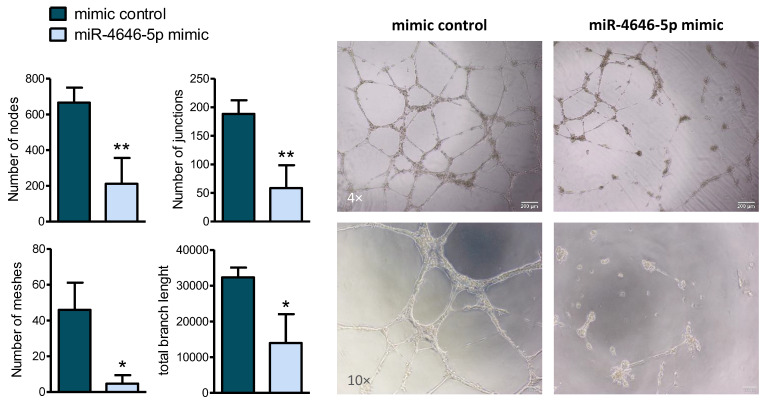
MiR-4646-5p reduces in vitro tube formation of endothelial cells. An in vitro tube formation assay was performed with human umbilical vein endothelial cells (HUVECs) cultured in an extracellular matrix to assess the impact of miR-4646-5p mimic transduction on branch formation. Representative images are shown (in two different magnifications on the (**right**), as well as the quantified number of nodes, junctions, meshes, and the total tube length (**left**) (*n* = 3; mean ± SD; * *p* ≤ 0.05, ** *p* ≤ 0.01).

Angiogenesis, the formation of new blood vessels, is a prerequisite for tumors to expand in size and to eventually invade the bloodstream to disseminate to other organs [35,36]. Numerous miRNAs have already been identified to affect angiogenesis in breast cancer [37]. Thus, we investigated the impact of miR-4646-5p on angiogenesis in vitro by assessing the branching tube formation of endothelial cells cultured in an extracellular matrix. For this purpose, human endothelial umbilical vein endothelial cells (HUVECs) were transiently transfected with the miR-4646-5p mimic (Supplementary Figure S6A), which resulted in a reduced ability of the cells to form tubes, as indicated by a reduction in meshes, nodes, junctions, and total branch length (Figure 4). In addition, amongst a panel of angiogenesis-related genes screened by RT-qPCR, we found three of those genes, namely fibroblast growth factor 2 (*FGF2*), interleukin 6 (*IL6*), and neuropilin-2 (*NRP2*), to be downregulated in HUVECs upon mimic transfection (Supplementary Figure S6B). This finding further substantiates potential anti-angiogenic properties of miR-4646-5p.

### 2.4. GRAM Domain-Containing Protein 1B (GRAMD1B) Is a Direct Target of miR-4646-5p and Affects the Growth of TNBC Cells

**Figure 5:**
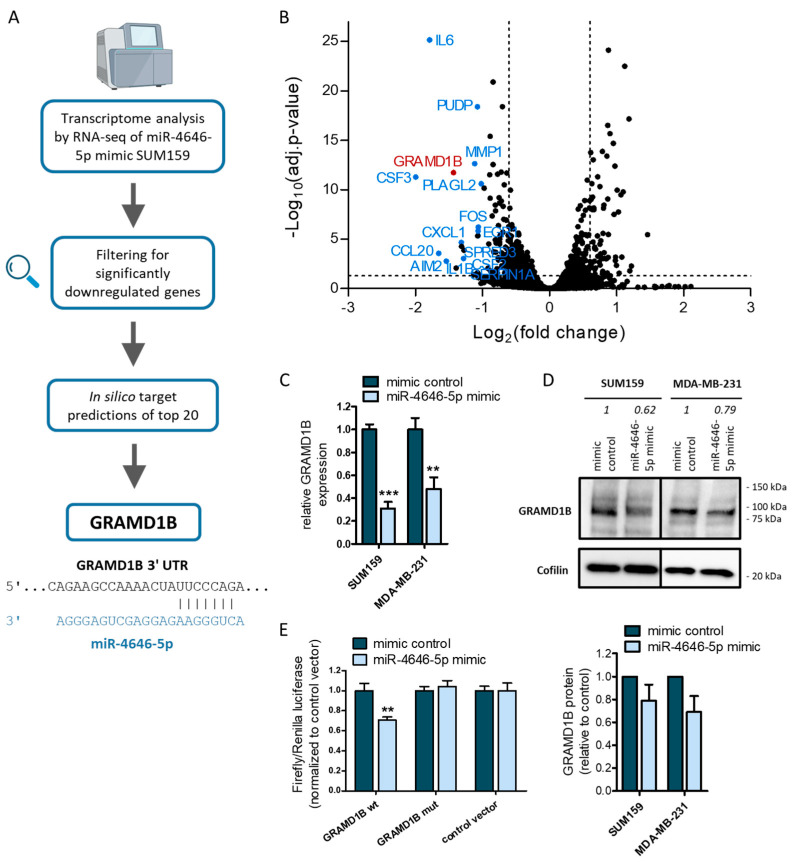
GRAM Domain-Containing Protein 1B (GRAMD1B) is a direct target of miR-4646-5p. (**A**) Scheme illustrating the identification of GRAMD1B by RNA-seq analysis of SUM159 miR-4646-5p mimic cells and in silico target predictions. At the bottom, a confirmed binding sequence between GRAMD1B and miR-4646-5p is presented. (**B**) Volcano plot of the miR-4646-5p mimic vs. mimic control RNA-seq results of SUM159 cells. The adjusted *p*-value cut-off of 0.05 for significantly deregulated genes and the 1.5-fold change thresholds are indicated as dashed lines. The top downregulated annotated genes, according to fold change, are highlighted and labeled. (**C**) The downregulation of GRAMD1B mRNA by the miR-4646-5p mimic was confirmed in two TNBC cell lines by RT-qPCR (*n* = 3; mean ± SD; ** *p* ≤ 0.01, *** *p* ≤ 0.001). (**D**) The downregulation of GRAMD1B protein by the miR-4646-5p mimic was confirmed in two TNBC cell lines by Western blotting (representative blot on top with the fold changes in signal intensity normalized to Cofilin and relative to the mimic controls indicated, and quantification of *n* = 3 blots at the bottom). (**E**) A dual luciferase reporter assay was performed, giving evidence for the direct binding of the miR-4646-5p mimic to a reporter carrying the wildtype (wt) GRAMD1B 3′ UTR target sequence, whereas no binding occurred to a mutated binding sequence (mut) or empty control vector (luciferase signals were normalized to the control vector) (*n* = 3; mean ± SD; ** *p* ≤ 0.01).

As our in vitro data had identified miR-4646-5p as a novel factor affecting the growth, proliferation, and migration of TNBC cells, we next sought to explore the molecular mechanisms behind these phenotypes. For this purpose, we conducted a whole transcriptome analysis of miR-4646-5p mimic-transfected SUM159 cells by RNA-seq. We found 605 genes that were significantly differentially expressed when compared to mimic control cells (277 upregulated and 328 downregulated; Figure 5B; Supplementary file RNAseq_sign. deregulated genes.xlsx). To identify potential direct targets of miR-4646-5p (an illustration of the target identification process is given in Figure 5A), we filtered the results and focused on downregulated genes, as this is the primary mechanism of action of miRNAs. We ranked the downregulated genes according to their fold change and ended up with a list of 83 annotated genes that were more than 1.5-fold downregulated, 15 of which were more than 2-fold downregulated. The top 20 downregulated genes are shown in Supplementary Table S2.

With the top 20 downregulated genes, in silico target predictions were conducted using three different algorithms (TargetScan [38], miRWalk2.0 [39], and miRDB [40]) to look for putative miRNA binding sites. For candidate genes, where at least two of the three algorithms predicted a binding site, the literature was searched to evaluate their possible function in cancer. Among the potential target candidates, the most promising was the GRAM Domain-Containing Protein 1B (GRAMD1B), as it had the highest number of predicted binding sites (a list of the predicted binding sites is given in Supplementary Table S3). GRAMD1B is an endoplasmic reticulum (ER)-anchored cholesterol transport protein that facilitates non-vesicular transport of accessible cholesterol from the plasma membrane to the ER, thereby contributing to cholesterol homeostasis [41,42]. Previous studies by Khanna et al. [43,44] also reported an influence of GRAMD1B on JAK/STAT and AKT signaling in cancer. We could not, however, confirm such an impact in our TNBC cell lines (Supplementary Figure S7B,C).

To determine whether GRAMD1B is indeed a target of miR-4646-5p, we first confirmed the downregulation of GRAMD1B on mRNA (Figure 5C) and protein level (Figure 5D, Supplementary Figure S8A) upon miR-4646-5p mimic transfection by RT-qPCR and Western blotting, respectively. Inhibition of miR-4646-5p had the opposite effect on GRAMD1B expression (Supplementary Figure S8B). Next, we proved the direct interaction of miR-4646-5p with one of the binding sites in the 3′ UTR of GRAMD1B (binding site 3 in Supplementary Table S3) in a dual luciferase reporter assay. Transfection of the miR-4646-5p mimic led to a reduced luciferase signal of the reporter construct carrying the GRAMD1B wildtype (wt) sequence, whereas the effect was abolished when the miR-4646-5p binding site was mutated (Figure 5E).

**Figure 6:**
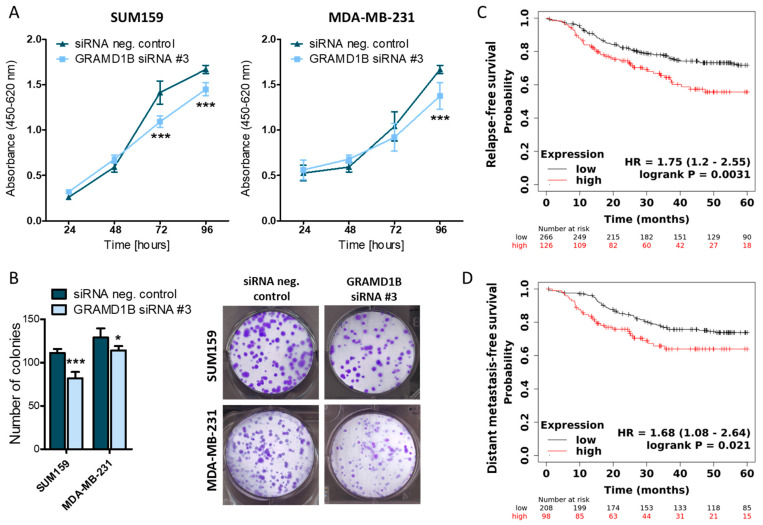
Knockdown of GRAMD1B reduces growth of TNBC cell lines, and higher levels of GRAMD1B are associated with worse patient survival. (**A**) The effect of siRNA-mediated GRAMD1B knockdown on cell growth of two TNBC cell lines was assessed by WST-1 assays (*n* = 6; mean ± SD; *** *p* ≤ 0.001). (**B**) The impact of siRNA-mediated knockdown of GRAMD1B on colony formation was observed in two TNBC cell lines. Absolute numbers of colonies were counted (representative images on the right) and are presented as mean ± SD (*n* = 6; * *p* ≤ 0.05; *** *p* ≤ 0.001). (**C**) Relapse-free survival of patients with TNBC split into a GRAMD1B low- (black; *n* = 266) and high-expression (red; *n* = 126) group based on microarray expression data (log-rank test; *p* = 0.0031; HR—Hazard Ratio). (**D**) Distant metastasis-free survival of patients with TNBC split into a GRAMD1B low- (black; *n* = 208) and high-expression (red; *n* = 98) group based on microarray expression data (log-rank test; *p* = 0.021; HR—Hazard Ratio).

To evaluate whether the downregulation of GRAMD1B by miR-4646-5p could contribute to the phenotype caused by the miRNA, we investigated the impact of siRNA-mediated knockdown of GRAMD1B (Supplementary Figure S7A,B) on the growth of SUM159 and MDA-MB-231 cells. The knockdown of GRAMD1B caused a decrease in cellular growth in WST-1 assays as well as a reduction in colony numbers in colony formation assays (Figure 6A,B). Importantly, this phenocopies the effect on growth caused by the miR-4646-5p mimic.

To further assess the role of GRAMD1B in TNBC, we performed a survival analysis of TNBC patients based on gene chip data from the Gene Expression Omnibus (GEO) database using a Kaplan–Meier analysis tool [45], which revealed that higher expression of GRAMD1B is associated with a significantly lower probability of relapse-free survival (log-rank test; *p* = 0.0031; Hazard ratio = 1.75; 95% confidence interval 1.2–2.55) and distant metastasis-free survival (log-rank test; *p* = 0.021; Hazard ratio = 1.68; 95% confidence interval 1.08–2.64) (Figure 6C,D). In summary, these results indicate that GRAMD1B itself may act as an oncogenic factor in TNBC and can thus partly explain the tumor-suppressive effects of miR-4646-5p, which we discovered to directly bind to and downregulate GRAMD1B mRNA.

### 2.5. MiR-4646-5p Has Pathway-Spanning Effects on Cholesterol Biosynthesis and Cytokine Signaling in TNBC Cells

**Figure 7:**
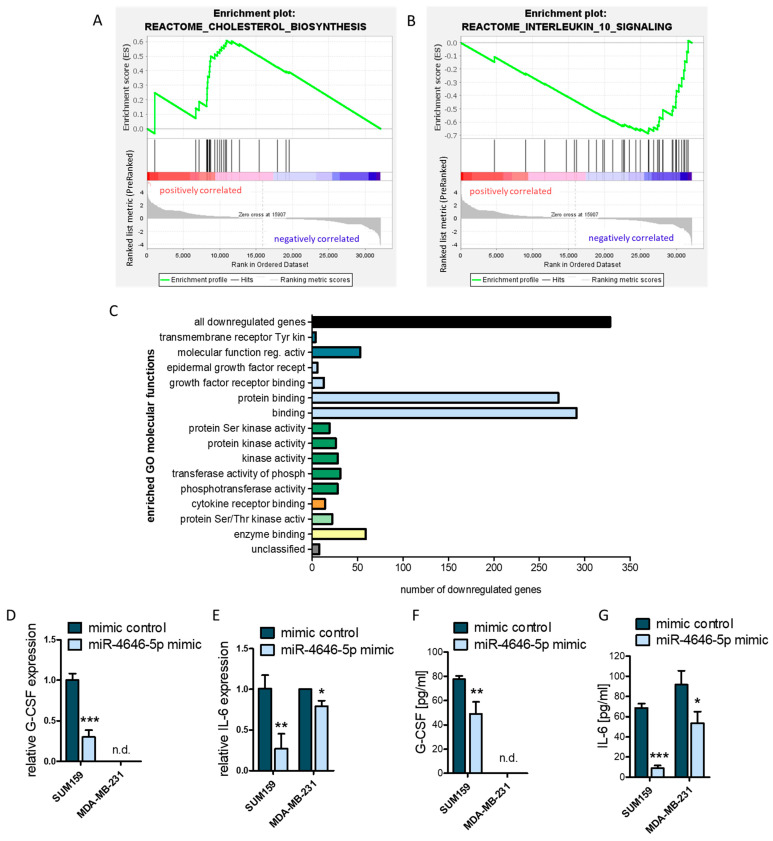
RNA-seq enrichment analysis and ELISAs show impact of miR-4646-5p on cholesterol biosynthesis as well as cytokine signaling and secretion. Gene set enrichment analysis of RNA-seq data from miR-4646-5p mimic-transfected SUM159 showed enrichment of (**A**) upregulated genes in the cholesterol biosynthesis pathway (normalized enrichment score = 1.52; *p* = 0.0299) and (**B**) downregulated genes in the interleukin-10 signaling pathway (normalized enrichment score = −2.02; *p* < 0.001). (**C**) PANTHER overrepresentation test of genes downregulated by miR-4646-5p. Depicted are all gene ontology (GO) molecular functions with significant (false discovery rate *p*-value < 0.05, Fisher’s Exact test) overrepresentations of downregulated genes and the number of downregulated genes in each GO group. Expression of (**D**) granulocyte colony-stimulating factor (G-CSF) and (**E**) interleukin-6 (IL-6) after miR-4646-5p mimic transfection of SUM159 and MDA-MB-231 cells, as measured by RT-qPCR (*n* = 3; mean ± SD; * *p* ≤ 0.05, ** *p* ≤ 0.01, *** *p* ≤ 0.001; n.d. = not detected). (**F**) G-CSF and (**G**) IL-6 ELISAs of the supernatant of miR-4646-5p mimic- or control-transfected SUM159 and MDA-MB-231 cells (*n* = 4; mean ± SD; * *p* ≤ 0.05, ** *p* ≤ 0.01, *** *p* ≤ 0.001; n.d. = not detected).

The phenotype that is caused by the deregulation of a miRNA is not based on a single target but its cumulative effect on possibly hundreds of direct as well as indirect targets and signaling networks [46]. Trying to ascribe the function of a miRNA to an exact molecular mechanism by just looking at single targets thus only gives an incomplete picture [46]. Therefore, we also approached the problem from a broader perspective by looking at entire signaling pathways by gene set enrichment analysis (GSEA) of our RNA-seq data. We discovered that miR-4646-5p induced a positive enrichment of upregulated genes in the cholesterol biosynthesis pathway (Figure 7A). As GRAMD1B is involved in cholesterol homeostasis, this result further corroborates that GRAMD1B is a target of miR-4646-5p. Moreover, GSEA showed that miR-4646-5p downregulated components of the interleukin-10 (IL-10) signaling pathway (Figure 7B). In addition to GSEA, we also performed a gene ontology (GO) overrepresentation analysis of the genes downregulated by miR-4646-5p. This revealed that the downregulated genes are overrepresented in molecular functions like growth factor receptor binding, serine and threonine receptor kinase activity, and, interestingly, cytokine receptor binding (Figure 7C, Supplementary Table S4), which is in line with the growth reductions we observed as well as with the GSEA finding of downregulated IL-10 signaling.

Taking a closer look at the list of genes that were downregulated by miR-4646-5p (Supplementary Table S2), it becomes apparent that many of the genes are, in fact, cytokines and chemokines involved in inflammation and tumor immunity. We selected the top two downregulated cytokines from the RNA-seq data, namely granulocyte colony-stimulating factor (G-CSF or CSF3) and interleukin-6 (IL-6), both of which have been reported to support breast cancer growth in various ways [47,48,49,50,51,52], and confirmed their downregulation by RT-qPCR. In the case of G-CSF, we could only confirm its downregulation by the miR-4646-5p mimic in the SUM156 cell line (which is in line with the RNA-seq data of this cell line) but did not detect any expression in MDA-MB231 cells (Figure 7D). IL-6 expression was detectable in both cell lines and showed strong downregulation upon miR-4646-5p mimic transfection (Figure 7E). To substantiate the possibility that miR-4646-5p might thereby also alter the secretome of TNBC cells, which could affect the tumor microenvironment, we next performed ELISAs. In accordance with the expression data, MDA-MB-231 cells did not secrete G-CSF, whereas in SUM159 cells, the miR-4646-5p mimic caused a significant reduction in the secretion of the cytokine (Figure 7F). IL-6 was secreted and markedly reduced by miR-4646-5p in both cell lines (Figure 7G).

## 3. Discussion

In this study, we characterized miR-4646-5p, a miRNA whose function in breast cancer had not yet been examined when it was first detected in an expression screen of three-dimensional breast cancer spheres [19]. An initial survival analysis of TCGA data revealed that higher expression of *miR-4646* was linked to significantly better overall survival of TNBC patients. This gave us reason to further explore the role of the miRNA in TNBC cell lines by using a miRNA mimic to facilitate overexpression as well as a complementary locked nucleic acid (LNA) antisense inhibitor. Both methodologies, miRNA mimics and antisense oligonucleotides, are being explored as miRNA-based therapeutics for the treatment of various types of cancer, including breast cancer [24,25,26].

We found that ectopic overexpression of miR-4646-5p led to reduced growth of TNBC cells, which seems to be connected to effects on proliferation and, depending on the cellular context, also the induction of apoptosis. The cell line context dependence of apoptosis induction by miR-4646-5p that we observed may generally be explained by the fact that the net effects a miRNA causes are based on a plethora of direct and indirect targets [33]. These targets can differ between different tissues and even between different cell lines of the same tissue, on the one hand, due to mutations or polymorphisms in target binding sites, and on the other hand, due to inherent differences in gene expression [33,53]. We showed that the two tested TNBC cell lines, SUM159 and MDA-MB-231, exhibit differences in the expression of pro-apoptotic genes. Moreover, it is known that the two cell lines have different mutational profiles [54], which may also influence the effect of miR-4646-5p on apoptosis.

In addition to the effect on growth, we observed that miR-4646-5p overexpression resulted in decreased migration, whereas inhibition had the corresponding opposite effect. Substantiating the migration-reducing effect of miR-4646-5p, our RNA-seq data showed a significant downregulation of three Rho guanine nucleotide exchange factors (GEFs), namely ArfGAP with RhoGAP domain, ankyrin repeat and PH domain 3 (*ARAP3*) (1.4-fold downregulated), Rho/Rac guanine nucleotide exchange factor 2 (*ARHGEF2*) (1.3-fold downregulated), and pleckstrin homology and RhoGEF domain-containing G4 (*PLEKHG4*) (1.3-fold downregulated). GEFs catalyze the activation of the members of the Rho GTPase family, which constitute central players in actin cytoskeleton remodeling and focal cell adhesion [55,56]. Both ARAP3 and ARHGEF2 have been associated with increased migration, invasion, and metastatic behavior in various cancer types [57,58,59,60,61]. The downregulation of these migration- and metastasis-associated factors may thus help to partly explain the reduced migration we observed upon miR-4646-5p mimic transfection.

The rearrangement of the actin cytoskeleton to form cell protrusions and the establishment of focal contacts with the extracellular matrix (ECM) are only the first steps required for cell migration [34]. These are followed by the recruitment of proteases, like matrix metalloproteinases (MMPs), to degrade the ECM [34]. In our RNA-seq data, we observed a 2.2-fold downregulation (12th most downregulated gene) of *MMP1* (collagenase), which was reported to be highly expressed in TNBC tissue and to be associated with migration, invasiveness, and metastasis of TNBC [62,63]. While the TargetScan algorithm [38] did predict one potential binding site, we did not confirm the direct interaction of miR-4646-5p with the MMP1 mRNA.

For tumors to be able to metastasize, not only is a migratory and invasive phenotype required, but another prerequisite is the formation of new blood vessels in tumors that enable oxygen supply and the dissemination of the primary tumor to distant organs [35,36]. We observed reduced tube formation and branching of endothelial HUVECs upon miR-4646-5p overexpression, indicating that miR-4646-5p might be able to act as an anti-angiogenic factor. Moreover, we found that miR-4646-5p reduces the mRNA levels of fibroblast growth factor 2 (*FGF2*), interleukin-6 (*IL6*), and neuropilin 2 (*NRP2*) in endothelial cells, all three of which are known to contribute to endothelial cell proliferation, migration, and vascularization [64,65,66]. None of the three was predicted to have binding sites for miR-4646-5p, but their indirect downregulation could explain the negative effect that the miRNA has on the tube formation of endothelial cells. Moreover, this provides a rationale for a therapeutic delivery approach of miR-4646-5p, as the miRNA does not only have suppressive effects on the tumor cells themselves but could potentially also inhibit angiogenesis by acting on endothelial cells.

In order to identify a direct target of miR-4646-5p, we focused on the top downregulated genes in our RNA-seq data and screened them for potential binding sites. The top four downregulated genes (granulocyte colony-stimulating factor (*GCSF* or *CSF3*), interleukin 6 (*IL6*), C-C motif chemokine ligand 20 (*CCL20*), and absent in melanoma 2 (*AIM2*)) did not show canonical miR-4646-5p binding sites. However, we found that GRAM Domain-Containing Protein 1B (*GRAMD1B*), the fifth most downregulated gene, contained seven putative miR-4646-5p binding sites according to TargetScan [38] and miRDB [40] algorithms. Using luciferase reporter assays, we were able to confirm the direct interaction of miR-4646-5p with one of these binding sites. Moreover, we discovered that downregulation of GRAMD1B phenocopies the growth-reducing effect of miR-4646-5p, suggesting that the downregulation of GRAMD1B might contribute to the tumor-suppressive properties of miR-4646-5p. GRAMD1B is a protein that is anchored in the membrane of the endoplasmic reticulum (ER) and locates to contact sites between the ER and plasma membrane (PM), where it senses the presence of accessible cholesterol in the PM via its GRAM domain [42,67,68]. In the case of an increase in accessible cholesterol, GRAMD1B transports the cholesterol to the ER via its StART-like domain [67,68,69]. To strictly regulate the uptake and de novo biosynthesis of cholesterol, the master transcriptional regulator sterol regulatory element-binding protein 2 (SREBP-2), which sits in the membrane of the ER, is deactivated in the presence of high cholesterol levels [68]. Hence, GRAMD1B supports overall cholesterol homeostasis [68,69]. In addition, it contributes to the production of steroid hormones, which are generated from cholesterol after its transport to the ER [69]. For example, Sandhu et al. showed that *GRAMD1B* knockout mice had reduced serum levels of corticosteroids, reduced cholesterol ester storage, and increased activity of SREBP-2 in the adrenal glands, the tissue with the highest expression of GRAMD1B, due to impaired PM-ER cholesterol transport [69]. Naito et al. described that the triple knockout of *GRAMD1A*, *GRAMD1B*, and *GRAMD1C* in HeLa cells caused an accumulation of accessible cholesterol in the PM and a reduced ability to inactivate SREBP-2 in response to cholesterol increase [67]. Similarly, a knockdown of GRAMD1B in macrophages was found to increase the pool of cholesterol in the PM and to cause higher expression of SREBP-2 target genes involved in cholesterol biosynthesis and uptake [70]. This correlates well with the fact that our RNA-seq analysis showed that upon miR-4646-5p transfection, which targets and downregulates GRAMD1B, genes in the cholesterol biosynthesis pathway were enriched among all upregulated genes.

Regarding the role of GRAMD1B in cancer, there are contradicting reports. GRAMD1B was first associated with taxane resistance in ovarian cancer [71]. Khanna et al. reported that GRAMD1B knockdown decreases STAT3 activation and expression of the anti-apoptotic factor Bcl-xL, thereby causing reduced growth and increased apoptosis induction in gastric cancer [43], which matches our observations in TNBC. However, the same authors then reported the opposite in the TNBC cell line MDA-MB-231, namely increased STAT3 and also AKT activation upon GRAMD1B knockdown, leading to increased migration [44]. We could not confirm any role of GRAMD1B in JAK/STAT3 or AKT signaling in TNBC cell lines. Based on our findings, we believe that GRAMD1B rather acts as an oncogenic factor in TNBC, as we found higher levels of GRAMD1B to be connected to significantly shorter relapse-free survival as well as distant metastasis-free survival of TNBC patients. Moreover, we showed that its downregulation reduced cell growth and colony formation of TNBC cells in vitro. These effects may be explained by its role in cholesterol transport. Due to their increased proliferation, cancer cells are in need of high levels of cholesterol for the formation of cell membranes, the production of steroid hormones, and the generation of different cholesterol metabolites, which have been found to promote tumor growth, like, for example, 6-oxo-cholestan-3β,5α-diol (OCDO) [72,73]. But de novo cholesterol synthesis is an energy- and time-consuming process, and in the presence of high levels of exogenous cholesterol, as is generally the case in cell culture medium in vitro, or due to a high-fat diet in vivo, cancer cells might favor the uptake of exogenous cholesterol over its synthesis [72]. To make use of exogenous cholesterol, it must be transported from the PM, where most of a cell’s cholesterol is located, to the ER [74]. It is well-established that GRAMD1B plays an essential role in this step, as downregulation of GRAMD1B was found to critically impair cholesterol transport from the PM to the ER [67,69,70]. Therefore, we speculate that upregulation of GRAMD1B, despite causing decreased SREBP-2 activation and cholesterol biosynthesis, would be beneficial for cancer cells, as it could support their increased uptake, storage, and use of exogenous cholesterol. The transport route from the PM to the ER via GRAMD1B is especially crucial for high-density lipoprotein (HDL)-derived cholesterol that enters the PM via uptake by the scavenger receptor class B, type I (SR-B1) [69,75]. In the case of breast cancer, numerous studies have shown that HDL exerts oncogenic effects. For example, Llaverias and colleagues showed that a cholesterol-rich diet enhanced tumor formation, growth, and metastasis in a breast cancer mouse model and that tumor-bearing mice had lower HDL serum levels and higher SR-B1 expression in tumors, indicating a higher consumption of HDL-derived cholesterol by the tumor cells [76]. Danilo et al. found that HDL is capable of stimulating proliferation and migration of breast cancer cell lines (both ER-positive and TNBC) and that a knockdown of SR-B1 was able to attenuate these effects [77]. There is evidence that GRAMD1B is not only involved in the transport of HDL-derived cholesterol but also low-density lipoprotein (LDL)-derived cholesterol, which is taken up via the LDL receptor (LDLR) into lysosomes and from there is distributed mostly directly to the ER [68,74]. Höglinger et al. reported that GRAMD1B localizes to lysosome–ER contact sites by interacting with Niemann–Pick type C protein 1 (NPC1) and thereby regulates cholesterol egress [78]. By doing so, GRAMD1B, in turn, might contribute to breast cancer, as LDL-derived cholesterol has been extensively described to promote the growth, proliferation, and migration of breast cancer cells, specifically of TNBC cells [79,80,81]. In summary, we believe that by targeting and downregulating GRAMD1B, miR-4646-5p exerts a tumor-suppressive effect, as it thereby might impair the transport and thus the use of exogenous cholesterol, which has been reported to constitute a vital pro-oncogenic source for breast cancer cells.

It is, however, clear that the tumor-suppressive effects of miR-4646-5p are not simply based on one direct target but on a plethora of direct as well as indirect targets, some of which were already discussed above. A closer look at the top downregulated genes in our RNA-seq analysis could provide further explanation for the tumor-suppressiveness of miR-4646-5p. For example, we found *FOS* proto-oncogene and early growth response factor 1 (*EGR1*) to be downregulated. Both function as transcription factors in the mitogen-activated protein kinase (MAPK) pathway, which is activated by external stimuli like growth factors and cytokines, and both were described to control tumor cell proliferation, apoptosis, migration, and invasion [82,83]. Fos, as part of the activator protein 1 (AP-1) transcription factor complex, also regulates the expression of various cytokines and chemokines, like IL-2, IL-4, IL-6, IL-8, interferon gamma (IFNγ), and tumor necrosis factor-alpha (TNFα) [84,85]. This draws a link to another observation, namely that the list of genes downregulated by miR-4646-5p includes numerous cytokines and chemokines that have been described to have tumor-promoting functions. The top two downregulated genes were the two cytokines *GCSF* and *IL6*. Not only did we observe their downregulation on the RNA level, but we also found their secretion to be reduced by miR-4646-5p on the protein level. G-CSF was reported to increase proliferation and growth of breast cancer cells, both by directly acting on the cancer cells as well as by recruiting immune cells, like myeloid-derived suppressor cells (MDSCs), which inhibit T cell activation and proliferation, thus enabling cancer immune evasion, and tumor-associated macrophages, which promote cancer cell growth [47,48,49]. Moreover, TNBC cells were found to express higher levels of G-CSF than ER-positive breast cancer cells, and high G-CSF expression was associated with poor overall survival of TNBC patients [49]. Autocrine IL-6 signaling, which proceeds via the JAK/STAT3 pathway, is known to promote breast cancer proliferation, survival, migration, and invasiveness [50]. Furthermore, IL-6 favors tumorigenesis by creating an immunosuppressive tumor microenvironment, as the induction of JAK/STAT3 signaling has an inhibitory effect on, for example, dendritic cells, effector T cells, and natural killer cells [52,86]. As both *GCSF* and *IL6* did not contain canonical binding sites for miR-4646-5p, their downregulation may have been mediated indirectly, for example, by the miRNA targeting transcription factors that control their expression. Other cytokines in the list of top downregulated genes were C-C motif chemokine ligand 20 (*CCL20*), C-X-C motif chemokine ligand 1 (*CXCL1*), as well as interleukin-1β (*IL1B*), which were also more than 2-fold downregulated by seemingly indirect mechanisms and which were reported to have diverse breast cancer-promoting properties [87,88,89,90].

To broaden the insight into the functions of miR-4646-5p in TNBC even further and to move away from the single-target perspective, we performed a gene set enrichment analysis which revealed downregulation of IL-10 signaling targets, namely tumor-promoting cytokines like *IL1A*, *IL1B*, *IL6*, *GCSF*, and *TNF*. A gene ontology (GO) overrepresentation analysis of the genes downregulated by miR-4646-5p also brought up cytokine binding as an overrepresented molecular function. Overall, this provides additional evidence for the pleiotropic effects of miR-4646-5p and hints at a role in cytokine signaling in TNBC cells. By downregulating these signaling molecules, miR-4646-5p could help to modulate the tumor microenvironment towards enhanced anti-tumor immunity. It might thus be of interest in the future to explore combinations of miR-4646-5p and immunotherapy, like checkpoint inhibitors, as therapeutic approaches.

## 4. Materials and Methods

### 4.1. In Silico Analysis and Patient Cohorts

To explore the prognostic significance of miR-4646-5p, its target GRAM Domain-Containing Protein 1B (*GRAMD1B*), and its host gene abhydrolase domain-containing 16A (*ABHD16A*), a publicly available Kaplan–Meier analysis tool was used (https://kmplot.com/analysis/index.php?p=service&cancer=breast_mirna, accessed on 30 October 2023 [31] for miR-4646-5p; https://kmplot.com/analysis/index.php?p=service&cancer=breast, accessed on 8 October 2023 [45] for *GRAMD1B* and *ABHD16A*) to analyze the overall survival of 97 patients with triple negative breast cancer (TNBC) (for miR-4646-5p), the relapse-free survival of 392 patients or distant metastasis-free survival of 306 patients with TNBC (for *GRAMD1B*), and the overall survival of 144 patients with TNBC or 296 patients with basal breast cancer (for *ABHD16A*). Patients were split into high- and low-expression groups by an auto-selected optimal cutoff. The dataset used for the analysis of miR-4646-5p was RNA-seq-based and originated from the Cancer Genome Atlas Project (TCGA) [31]. The analyses of *GRAMD1B* and *ABHD16A* were based on gene chip data from the Gene Expression Omnibus (GEO) database [45].

### 4.2. Cell Lines and Cell Culture Conditions

The two human triple negative breast cancer cell lines SUM159 and MDA-MB-231 were used in this study. MDA-MB-231 cells were purchased from the American Type Culture Collection (ATCC; Manassas, CA, USA). The SUM159 cell line was obtained from Asterand (Detroit, MI, USA). Furthermore, HEK239 cells were purchased from the American Type Culture Collection (ATCC), and single-donor human umbilical vein endothelial cells (HUVECs) were acquired from Lonza (Basel, Switzerland).

MDA-MB-231 and HEK239 cells were cultured in high-glucose DMEM (4.5 g/L D-Glucose, L-Glutamine, 25 mM HEPES; Gibco, Thermo Fisher Scientific, Waltham, MA, USA), 10% fetal bovine serum (FBS) (Serana, Pessin, Germany), and 1% penicillin/streptomycin (final concentration penicillin: 100 units/mL, final concentration streptomycin: 100 µg/mL; Sigma-Aldrich, St. Louis, MO, USA). SUM159 cells were maintained in Ham’s Nutrient Mixture F12 containing 1 mM L-Glutamine (GE Healthcare Life Sciences, Pittsburgh, PA, USA) and 2 mM HEPES buffer (Gibco), 5 μg/mL insulin (Sigma-Aldrich), 1 μg/mL hydrocortisone (Sigma-Aldrich), 1% penicillin/streptomycin (Sigma-Aldrich), and 5% FBS (Serana). HUVECs were cultured in EBMTM-2 Basal Medium (Lonza) supplemented with EGMTM-2 SingleQuots^TM^ Supplements (Lonza). All cell lines were cultivated at 37 °C in a 5% CO_2_ humidified incubator. Mycoplasma tests were run on the SUM159 and MDA-MB-231 cell line with the Venor^®^ GeM Mycoplasma Detection Kit (Minerva Biolabs, Berlin, Germany) by the Core Facility for Alternative Biomodels and Preclinical Imaging of the Medical University of Graz, Austria.

### 4.3. Transient miR-4646-5p Mimic/Inhibitor Transfection and Transient GRAMD1B Knockdown

For transient overexpression or inhibition of miR-4646-5p, cells were transfected with 10 nM mirVana™ hsa-miR-4646-5p mimic and mirVana™ mimic control (Thermo Fisher Scientific) or 50 nM hsa-miR-4646-5p miRCURY LNA miRNA Inhibitor and miRCURY LNA miRNA Inhibitor Control A (Qiagen, Venlo, The Netherlands), respectively. For transient knockdown of GRAMD1B, cells were transfected with 40 nM GRAMD1B siRNA #3 (targeting sequence AGGAATCGCTATCATTGACAA; Qiagen) or AllStars Negative Control siRNA (Qiagen). As a positive apoptosis control, cells were transiently transfected with 10 nM AllStars Hs Cell Death Control siRNA (Qiagen).

Cells in 6-well plates and 6 cm dishes were transfected with HiPerFect Transfection Reagent (Qiagen) according to the fast-forward protocol of the manufacturer. Cells in 96-well plates were transfected following the reverse transfection protocol. Overexpression/inhibition of miR-4646-5p and knockdown of GRAMD1B were confirmed by quantitative RT-PCR (RT-qPCR) 48 h after transfection.

### 4.4. Generation of Stable SUM159 miR-4646-5p Inhibitor Cells by Lentiviral Transduction

SUM159 cells were seeded in a 24-well format, and after 24 h, medium was replaced with complete growth medium containing ViralPlus Transduction Enhancer (ABM, Richmond, BC, Canada) diluted 1:200 and 10 μg/mL polybrene (Santa Cruz Biotechnology, Santa Cruz, CA, USA). A total of 10 μL of miR-4646-5p inhibitor virus (LentimiRa-Off-hsa-miR-4646-5p Virus, ABM) or control virus (Lenti-III-mir-Off Control Virus, ABM) were added dropwise. Forty-eight hours after transduction, selection was started using 1.5 μg/mL puromycin dihydrochloride (Gibco) and continued for one week while monitoring GFP expression by fluorescent microscopy. Cells were sorted by fluorescent-activated cell sorting for high GFP expression.

### 4.5. RNA Isolation and cDNA Synthesis

For RT-qPCR, RNA was isolated from fresh cells (at a confluency of 75 to 95%) in biological triplicates using TRIzol™ Reagent (Thermo Fisher Scientific) according to the manufacturer’s protocol. For each sample, 1 μg RNA was reverse transcribed into cDNA either using the miScript II RT Kit (Qiagen) with the miScript HiFlex Buffer for the detection of both miRNA and mRNA or using the QuantiTect Reverse Transcription Kit (Qiagen) for the detection of mRNA, both according to the manufacturer`s protocols.

### 4.6. Quantitative RT-PCR (RT-qPCR)

RT-qPCR was performed in technical duplicates with the QuantiTect SYBR Green PCR Kit (Qiagen) following the manufacturer’s two-step RT-PCR protocol. The following primers were used:

The Hs_miR-4646-5p_1 miScript Primer Assay (Qiagen) together with the miScript Universal Primer from the miScript SYBR Green PCR Kit (Qiagen). MiR-4646-5p expression was normalized to the two housekeepers *SNORD61* and *SNORD95* using the Hs_SNORD61_11 and Hs_SNORD95_11 miScript Primer Assays (Qiagen) according to the manufacturer`s instructions.

Primers for the detection of coding genes and the two corresponding housekeepers *GAPDH* and *U6* were designed with the NIH Primer Blast Tool (https://www.ncbi.nlm.nih.gov/tools/primer-blast/) and ordered from Eurofins Scientific (Luxembourg). A list of these primer sequences is given in Supplementary Table S1.

Per RT-qPCR reaction, 1 ng of cDNA was applied in a 10 µL reaction volume. Measurements were conducted in LightCycler^®^ 480 Multiwell Plates 384 on a LightCycler^®^ 480 Real-Time PCR System (Roche, Basel, Switzerland). For Ct value normalization, the arithmetic means of the according housekeeper genes were subtracted to receive delta Ct (ΔCt) values. Relative expression levels were calculated by subtracting the ΔCt of the respective negative control and were plotted as 2^−ΔΔCt^.

### 4.7. WST-1 Cell Growth Assay

To study the effect of miR-4646-5p and GRAMD1B on cell growth, WST-1 assays (Roche) were performed. For this purpose, 3 × 10^3^ SUM159 or 5 × 10^3^ MDA-MB-231 cells were seeded per well of a 96-well plate (one plate per time point) and transiently transfected using HiPerFect Transfection Reagent (Qiagen) according to the reverse transfection protocol of the manufacturer. Cells were cultivated for 24, 48, 72, and 96 h, and at each time point, WST-1 proliferation reagent (Roche) was added to each well in a 1:10 ratio and subsequently incubated at 37 °C for 60 or 120 min (depending on signal intensity). Colorimetric changes were measured using a SPECTROstar Omega (BMG LabTech, Ortenberg, Germany) at a wavelength of 450 nm with a reference wavelength of 620 nm.

### 4.8. Colony Formation Assay

To investigate the impact on cell growth over a longer time, clonogenic assays were used. Twenty-four hours after transfection, transiently transfected cells were trypsinized, counted, and seeded in 6-well plates (200 cells/well for SUM159, 500 cells/well for MDA-MB-231). Cells were incubated at standard conditions, and after 7 or 14 days (depending on the cell line), cells were fixed and stained with 0.04% crystal violet (Sigma-Aldrich) in 20% methanol/PBS. Colonies were counted manually, and each experiment was carried out in triplicates or sextuplicates.

### 4.9. EdU Proliferation Assay

The flow cytometric Click-iT™ Plus EdU Pacific Blue™ Flow Cytometry Assay Kit (Thermo Fisher Scientific) was used to assess cell proliferation. Cells were transfected in 6 cm dishes with HiPerfect (Qiagen) according to the fast-forward protocol of the manufacturer. Before harvesting, cells were labeled with 10 µM EdU in growth medium for 2 h at 37 °C and 5% CO_2_. All further steps were performed following the Click-iT™ Plus EdU Pacific Blue™ Flow Cytometry Assay protocol. Samples were run on a CytoFLEX SI (Beckman Coulter, Brea, CA, USA), recording 20,000 events (in R1) per sample. Gates were set to exclude debris/dead cells (R1) and cell aggregates (R2).

### 4.10. Caspase-3/7 and Caspase-9 Activity Assay

To measure the activity of the two apoptotic effector caspases caspase-3 and -7, and of caspase-9, Caspase-Glo^®^ 3/7 assays (Promega, Madison, WI, USA) and Caspase-Glo^®^ 9 assays (Promega) were performed according to the manufacturer’s instructions. Cells were transiently transfected in 96-well plates with 3 × 10^3^ SUM159 and 5 × 10^3^ MDA-MB-231 cells per well using HiPerFect Transfection Reagent (Qiagen), as stated previously. Forty-eight or seventy-two hours after transfection, the luminogenic reagents were added as instructed in the protocols. Signals were measured using a LUMIstar Omega (BMG LabTech).

### 4.11. Protein Extraction and Western Blotting

Total protein was extracted from cells with Radioimmunoprecipitation Assay (RIPA) buffer (Sigma-Aldrich) supplemented with 1:50 protease inhibitor cocktail P8340 (Sigma-Aldrich). Per sample, 25 µg of protein was substituted with Laemmli buffer (BioRad, Hercules, CA, USA) containing 10% β-mercaptoethanol (Sigma-Aldrich). Proteins were separated by sodium dodecyl sulfate polyacrylamide gel electrophoresis (SDS-PAGE) on a 4–15% Mini-PROTEAN^®^ TGX™Precast Gel (BioRad) before plotting on nitrocellulose membranes (BioRad). Membranes were blocked in 5% non-fat milk powder/1× Tris-buffered saline (TBS; BioRad)/0.1% Tween-20 (Sigma-Aldrich). Membranes were incubated with primary antibodies overnight at 4 °C. The following primary antibodies were used (diluted in 5% Bovine serum albumin (BSA; Sigma-Aldrich)/TBS-Tween): PARP (#9542, Cell Signaling Technology, Danvers, MA, USA) diluted 1:1000, Cofilin (ab42824, Abcam, Cambridge, UK) diluted 1:10,000, GRAMD1B (Proteintech Europe, Manchester UK) diluted 1:1000, Akt (#9272, Cell Signaling Technology) diluted 1:1000, Phospho-Akt (Ser473) (D9E XP^®^ Rabbit mAb #4060, Cell Signaling Technology) diluted 1:2000, STAT3 (79D7 Rabbit mAb #4904, Cell Signaling Technology) diluted 1:5000, and Phospho-STAT3 (Tyr705) (D3A7 XP^®^ Rabbit mAb #9145, Cell Signaling Technology) diluted 1:1000. Following primary antibody incubation, membranes were washed in TBS-Tween (3 times for 10 min each), incubated with the secondary HRP-conjugated anti-rabbit antibody (diluted 1:1000 in 5% milk/TBS-Tween; Santa Cruz Biotechnology, Dallas, TX, USA) for 1 h, and washed again. Signals were detected using an enhanced chemiluminescence detection system (SuperSignal^TM^ West Pico Chemiluminescent Substrate, Thermo Fisher Scientific; or SuperSignal™ West Femto Maximum Sensitivity Substrate, Thermo Fisher Scientific) on a BioRad ChemiDoc Touch device. Densitometric quantifications were performed with the Image Lab Software Version 6.1.0 build 7 (BioRad) using volume tools. To re-probe membranes, they were stripped with 10% acetic acid for 1 h.

### 4.12. Scratch Assay

SUM159 cells were transiently transfected with the miR-4646-5p mimic or control in 6-well plates with four biological replicates. High cell numbers were seeded (5 × 10^5^) to reach confluence after 24 h, and scratches were introduced. Medium was changed, and cells were washed to remove scratched-off cells. Closure of the scratches was documented under the microscope after 15/20 h, 24 h, and 45 h. The ImageJ (NIH, Bethesda, MD, USA) plugin “MRI Wound Healing Tool” was used to determine the scratch areas, and for each time point, the remaining area relative to the 0 h time point was calculated.

### 4.13. Transwell Migration Assay

To confirm the scratch assay results, transwell migration assays were conducted using 0.4 µm pore size transwell membranes (Corning Incorporated, Corning, NY, USA). Cells were transiently transfected, and after 24 h, medium was changed to start starvation without FBS. After 24 h of starvation, cells were trypsinized and seeded on transwell membranes (1.5 × 10^4^ cells for SUM159, 2.5 × 10^4^ cells for MDA-MB-231). Membranes were previously coated with 0.1% gelatin (Sigma-Aldrich) in 0.02 M acetic acid, dried overnight, and re-hydrated with FBS-free medium at 37 °C for 1 h before seeding the cells in FBS-free medium. Lower chambers were filled with full growth medium containing FBS. After 48 h, cells were fixed with cold methanol and stained with 0.2% crystal violet (Sigma-Aldrich) in 2% ethanol. Cells that did not migrate through the membrane were removed. Microscopic images of five representative areas per transwell were taken, and migrated cells were counted.

### 4.14. Tube Formation Assay

In vitro tube formation of human umbilical vein endothelial cells (HUVECs) was assessed. HUVECs were transfected with 10 nM miR-4646-5p mimic or control in 6-well plates for 48 h before being seeded on an ECM matrix (ECM625; Merck, Darmstadt, Germany) in a 96-well plate with 1.5 × 10^4^ cells/well according to the manufacturer’s protocol. After 16 h, tube formation was observed under the microscope and quantified using the ImageJ plugin “Angiogenesis Analyzer” as described by Carpentier et al. [91].

### 4.15. Transcriptome Analysis and Identification of Potential miR-4646-5p Targets

For RNA-seq analysis, total RNA was isolated from fresh pellets of miR-4646-5p mimic/control-transfected SUM159 cells using the RNeasy Mini Kit with DNAse treatment according to the manufacturer’s instructions (Qiagen). After RNA quality control with an Agilent RNA 6000 Nano Kit on an Agilent 2100 BioAnalyzer system (Agilent Technologies, Santa Clara, CA, USA), and quantification using a NanoDrop 2000 Spectrophotometer (Thermo Fisher Scientific), 250 ng total RNA per sample was used for library preparation with the NEBNext^®^ rRNA Depletion Kit v2 and the NEBNext^®^ Ultra™ II Directional RNA Library Prep Kit for Illumina^®^ according to manufacturer’s instructions (New England Biolabs GmbH, Frankfurt am Main, Germany). Libraries were QC checked with an Agilent 2100 DNA high-sensitivity kit (Agilent Technologies), pooled, and sent to Vienna BioCenter Core Facilities GmbH (Vienna, Austria) for sequencing in an Illumina NovaSeq SP flow cell (Illumina, Eindhoven, Netherlands) in SR100 mode. After demultiplexing, FASTQ files were used for further analysis.

To identify potential miR-4646-5p targets, significantly downregulated genes were determined based on *p*-values adjusted for multiple testing and ranked according to log2-fold change values. For the top 20 downregulated genes, in silico target predictions were performed using three prediction algorithms (TargetScan [38], miRWalk2.0 [39], and miRDB [40]).

Gene set enrichment analysis (GSEA) was performed on the RNA-seq data using GSEA software release 3.0 (UC San Diego and Broad Institute, San Diego, CA, USA). In addition, a Gene Ontology (GO) enrichment analysis was conducted on significantly downregulated genes using the PANTHER Overrepresentation Test (http://www.pantherdb.org/; GO Ontology database https://doi.org/10.5281/zenodo.6799722, released 1 July 2022, reference list Homo sapiens, annotation data set GO molecular function complete, Fisher’s Exact Test).

### 4.16. Dual Luciferase Reporter Assay

To test for the direct interaction of miR-4646-5p with GRAMD1B, a 49 nt region of the 3′ UTR of GRAMD1B containing a predicted binding site was inserted into the dual luciferase reporter vector pEZX-MT06 (Genecopoeia, Rockville, MD, USA). In addition to the wild-type sequence (5′ GCACAGCCAGAAGCCAAAACTATTCCCAGAAAGTTTTG AATGCAAAACT 3′) a mutated sequence (5′ GCACAGCCAGAAGCCAAAACTATAGCTGGCAAGTTTTGAATGCAAAACT 3′) was also used. An empty control plasmid (CmiT000001-MT06; Genecopoeia) served as a reference control. HEK293 cells were seeded in 24-well plates and cultured until the cells reached 70–80% confluence. Cells were then co-transfected with 200 ng pEZ-MT06 GRAMD1B wt/mutated reporter vector or control vector and 50 nM mirVana™ miR-4646-5p mimic or mirVana™ mimic control (Thermo Fisher Scientific) or 50 nM hsa-miR-4646-5p miRCURY LNA miRNA Inhibitor and miRCURY LNA miRNA Inhibitor Control A (Qiagen), using Lipofectamine 2000 Transfection Reagent (Thermo Fisher Scientific) and Opti-MEM Reduced Serum Medium (Thermo Fisher Scientific) according to the manufacturer’s instructions. Cells were harvested 24 h after transfection and the Luc-Pair Luciferase Assay Kit 2.0 (Genecopoeia) was applied according to the user manual. Luminescence was measured using a LUMIStar Omega luminometer (BMG LabTech). The firefly luciferase signals were normalized to the renilla luciferase signals.

### 4.17. G-CSF and IL-6 ELISA

To determine the impact of miR-4646-5p on the secretion of the two cytokines granulocyte colony-stimulating factor (G-CSF) and interleukin-6 (IL-6), Enzyme-linked Immunosorbent Assays (ELISAs) were performed on the supernatant of SUM159 and MDA-MB-231 cells using the Human G-CSF Instant ELISA™ Kit (Invitrogen, Thermo Fisher Scientific) and IL-6 Human Instant ELISA™ Kit (Invitrogen, Thermo Fisher Scientific), respectively. Cells were transfected with 10 nM miR-4646-5p mimic or control, and after 24 h, growth medium was replaced by serum-free medium. After another 40 h, supernatant was harvested and cleared by centrifugation. A total of 50 μL supernatant was used per well in technical duplicates. Standards and blank were prepared according to the manufacturer’s instructions. After 3 h of incubation, wells were washed 6 times, and substrate solution was added according to instructions. Absorbances were read at 450 nM and 620 nM as reference using a SPECTROstar Omega spectrophotometer (BMG Labtech).

### 4.18. Statistical Analysis

Statistical analysis was performed using GraphPad Prism Version 5.01 (GraphPad Software, Inc., San Diego, CA, USA). Differences between samples and respective controls were assessed by unpaired, two-tailed independent t-tests with a 95% confidence interval, unless indicated otherwise. *P*-values below 0.05 were considered statistically significant (* *p* ≤ 0.05, ** *p* ≤ 0.01, *** *p* ≤ 0.001).

## 5. Conclusions

Based on the findings of this study, we conclude that miR-4646-5p acts as a tumor-suppressive factor in TNBC, as we observed the miRNA to decrease growth, colony formation, proliferation, and migration of TNBC cells. The mechanisms behind these phenotypes seem diverse, which was supported by our RNA-seq analysis showing the downregulation of a variety of tumor-promoting factors. Among these, we identified GRAMD1B as a direct target of miR-4646-5p, which supports TNBC growth probably due to its role in cholesterol transport. Moreover, miR-4646-5p not only exerts its effects in cancer cells but could potentially facilitate comprehensive tumor suppression. We discovered it to decrease tube formation of endothelial cells, possibly reducing angiogenesis in vivo, and to cause reduced expression as well as secretion of tumor-promoting cytokines, which could help modulate the tumor microenvironment towards increased anti-cancer immunity.

## Data Availability

All data relevant to or analyzed in the study are contained within the article or Supplementary Materials, or available from the corresponding author upon request.

## Supplementary Materials

The following supporting information can be downloaded at: https://www.mdpi.com/article/10.3390/ncrna10010002/s1, Supplementary Figures (Figures S1–S8), Supplementary Tables (Tables S1–S4), RNAseq_sign. deregulated genes.xlsx.
