# Supplementary material for: MiR-4646-5p Acts as a Tumor-Suppressive Factor in Triple Negative Breast Cancer and Targets the Cholesterol Transport Protein GRAMD1B
Source: Noncoding RNA. 2023 Dec 26;10(1):2. doi: 10.3390/ncrna10010002 (PMC10801495; doi:10.3390/ncrna10010002)
Supplement: Supplementary file 1 [file ncrna-10-00002-s001.zip › Supplementary Materials.pdf]

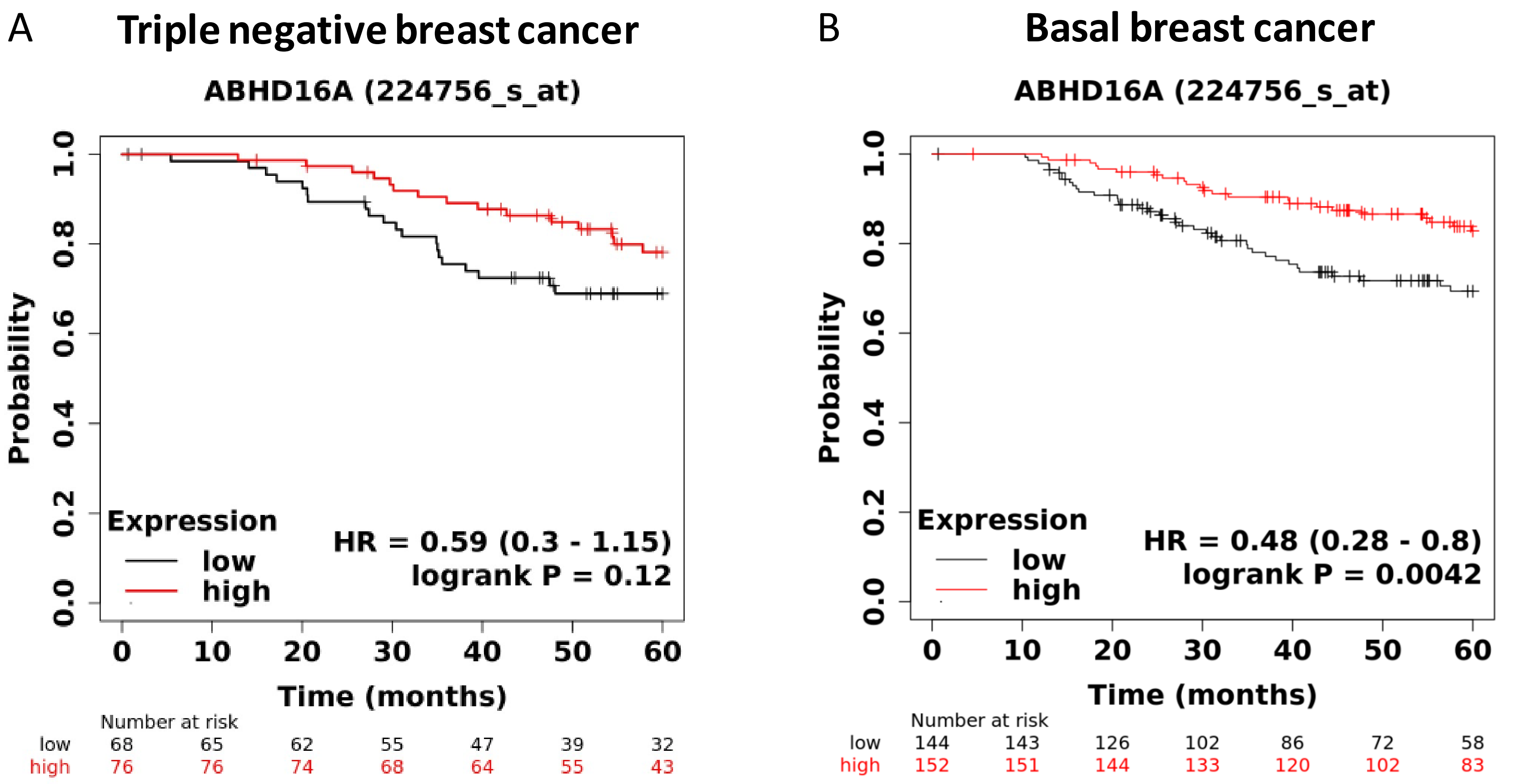

Supplementary Figure S1. Kaplan Meier curve analysis of the miR-4646-5p host gene abhydrolase domain containing 16A (ABHD16A). Overall survival of **(A)** patients with triple negative breast cancer split into an ABHD16A low (black; n = 68) and high (red; n = 76) expression group based on microarray data from the Gene Expression Omnibus (GEO) (log-rank test; p = 0.12; Hazard ratio = 0.59; 95% confidence interval 0.3 – 1.15) and **(B)** patients with basal breast cancer (PAM50 subtype classification) split into an ABHD16A low (black; n = 144) and high (red; n = 152) expression group based on GEO microarray data (log-rank test; p = 0.0042; Hazard ratio = 0.48; 95% confidence interval 0.28 – 0.8).

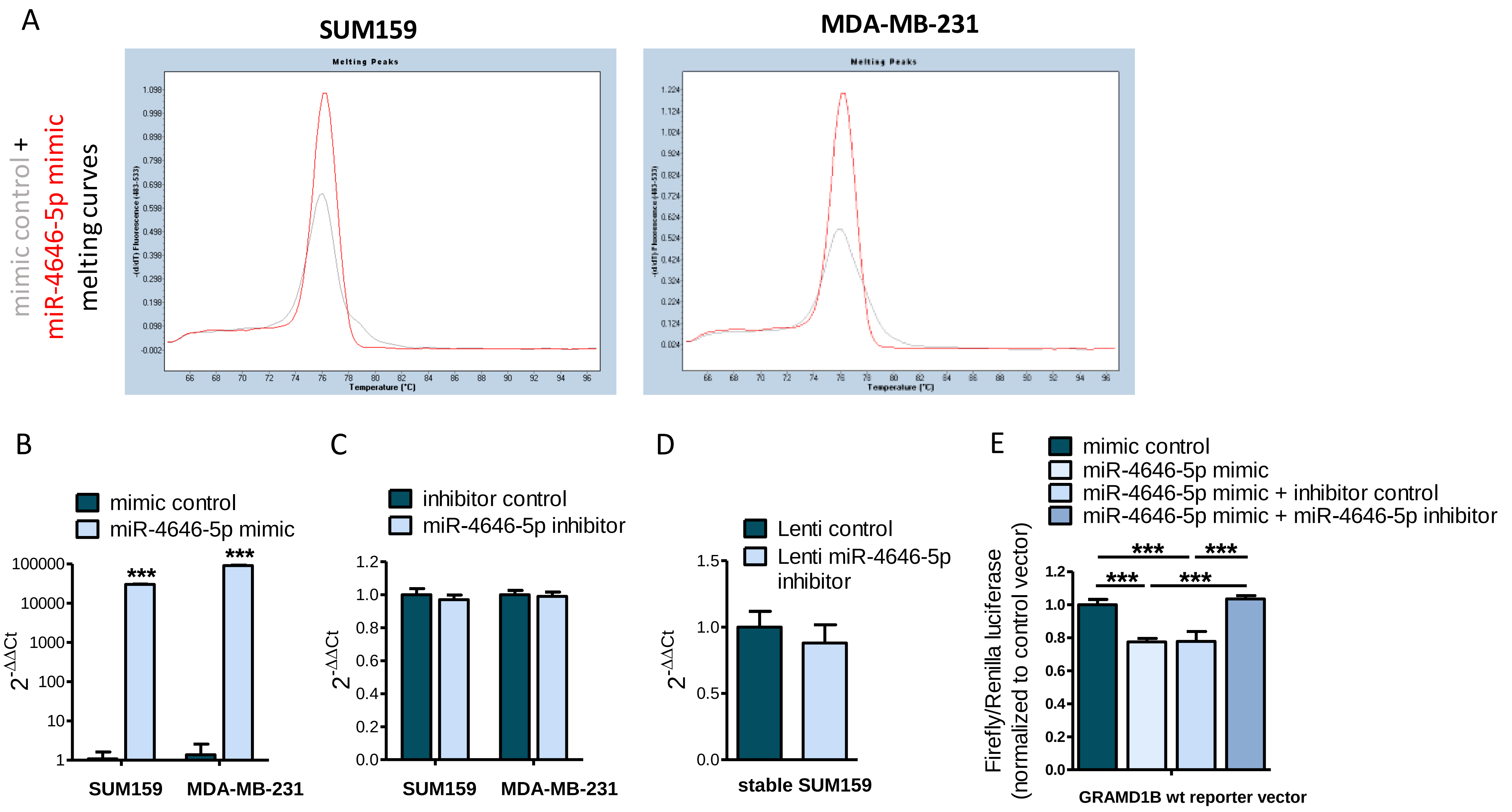

Supplementary Figure S2. MiR-4646-5p overexpression and inhibition in triple negative breast cancer (TNBC) cell lines. (A) Melting curve analysis of quantitative RT-PCR-based detection of miR-4646-5p in SUM159 and MDA-MB-231 cells transfected with miR-4646-5p mimic (red) or mimic control (gray) shows for the endogenous expression (control) a specific but weaker peak than for the overexpressed mimics **(B)** Transient overexpression of miR-4646-5p by mimic transfection (48 h) in two TNBC cell lines measured by RT-qPCR, depicted relative to the control (mean ± SD; n = 3; ***p ≤ 0.001). **(C, D)** Transient (48 h, left) and stable lentiviral (right) inhibition of miR-4646-5p in TNBC cell lines as determined by RT-qPCR, depicted relative to the respective controls (mean ± SD; n = 3). **(E)** Dual luciferase reporter assay using a reporter with a direct binding site of miR-4646-5p from the 3' UTR of the wildtype (wt) GRAM Domain-Containing Protein 1B gene (*GRAMD1B*). The reporter was co-transfected with miR-4646-5p mimic alone or in combination with the miR-4646-5p inhibitor or inhibitor control, showing that the specific inhibitor can bind and block the effect of the miRNA. The luciferase signals were normalized to the respective signals from an empty control vector (n = 3; mean ± SD; ***p ≤ 0.001).

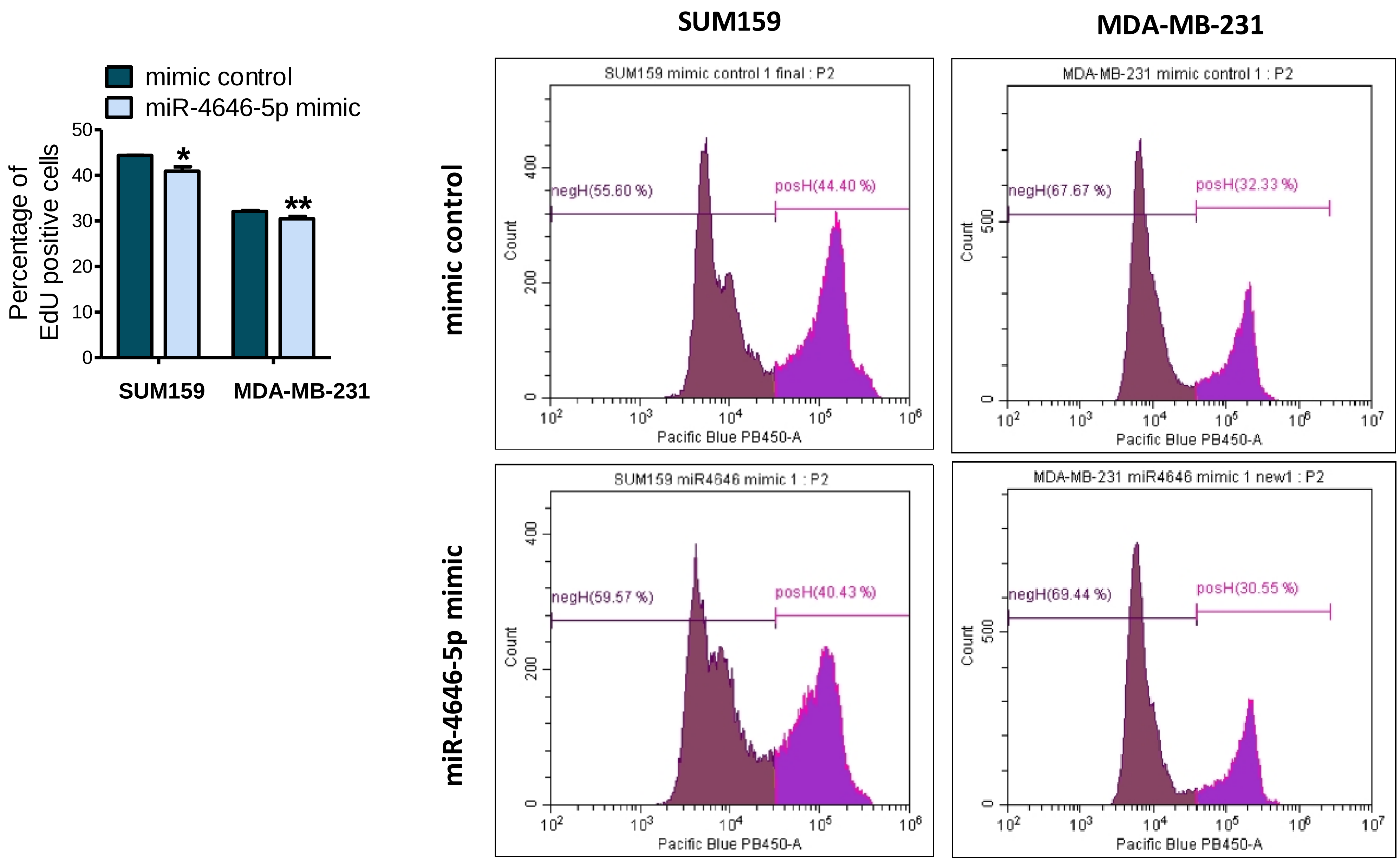

Supplementary Figure S3. MiR-4646-5p overexpression reduces proliferation of TNBC cell lines. Cell proliferation was assessed 72 h after transient miR-4646-5p mimic transfection by a flow cytometric EdU assay (n = 3; for SUM159 mimic control n = 2; mean ± SD; *p ≤ 0.05, **p ≤ 0.01). Representative histograms on the right show the distribution and gating of EdU negative and positive cells in the two TNBC cell lines.

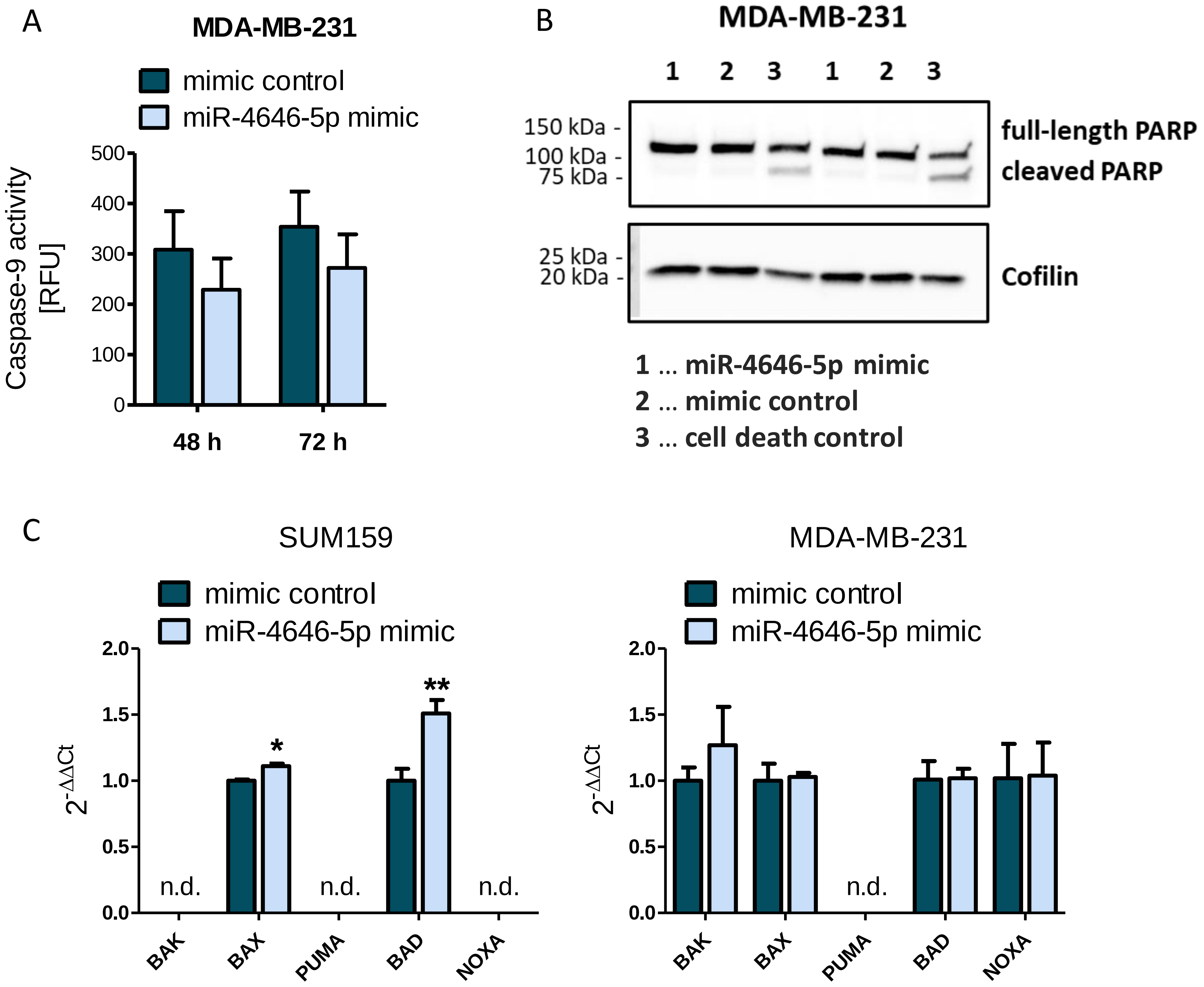

Supplementary Figure S4. Effect of miR-4646-5p overexpression on caspase-9 activity, PARP cleavage, and the expression of pro-apoptotic genes. (A) Luminescent caspase-9 activity assays were performed 48 h and 72 h after miR-4646-5p mimic or control transfection to investigate apoptosis induction in MDA-MB-231 cells (n = 6; mean ± SD; RFU = Relative fluorescence units). **(B)** The cleavage of PARP, a process indicative of apoptosis, was detected by Western blotting 48 h after transient miR-4646-5p mimic, negative mimic control, or positive cell death control transfection of MDA-MB-231 cells. **(C)** The expression of the pro-apoptotic genes BAK, BAX, PUMA, BAD, and NOXA was measured by qPCR 48 h after miR-4646-5p mimic or control transfection (n = 3; mean ± SD; *$p \leq 0.05$, **$p \leq 0.01$; n.d. = not detected). BAK…BCL2 homologous antagonist killer, BAX…BCL2 associated X, apoptosis regulator, PUMA…p53 upregulated modulator of apoptosis, BAD…BCL2 associated agonist of cell death, NOXA…phorbol-12-myristate-13-acetate-induced protein 1.

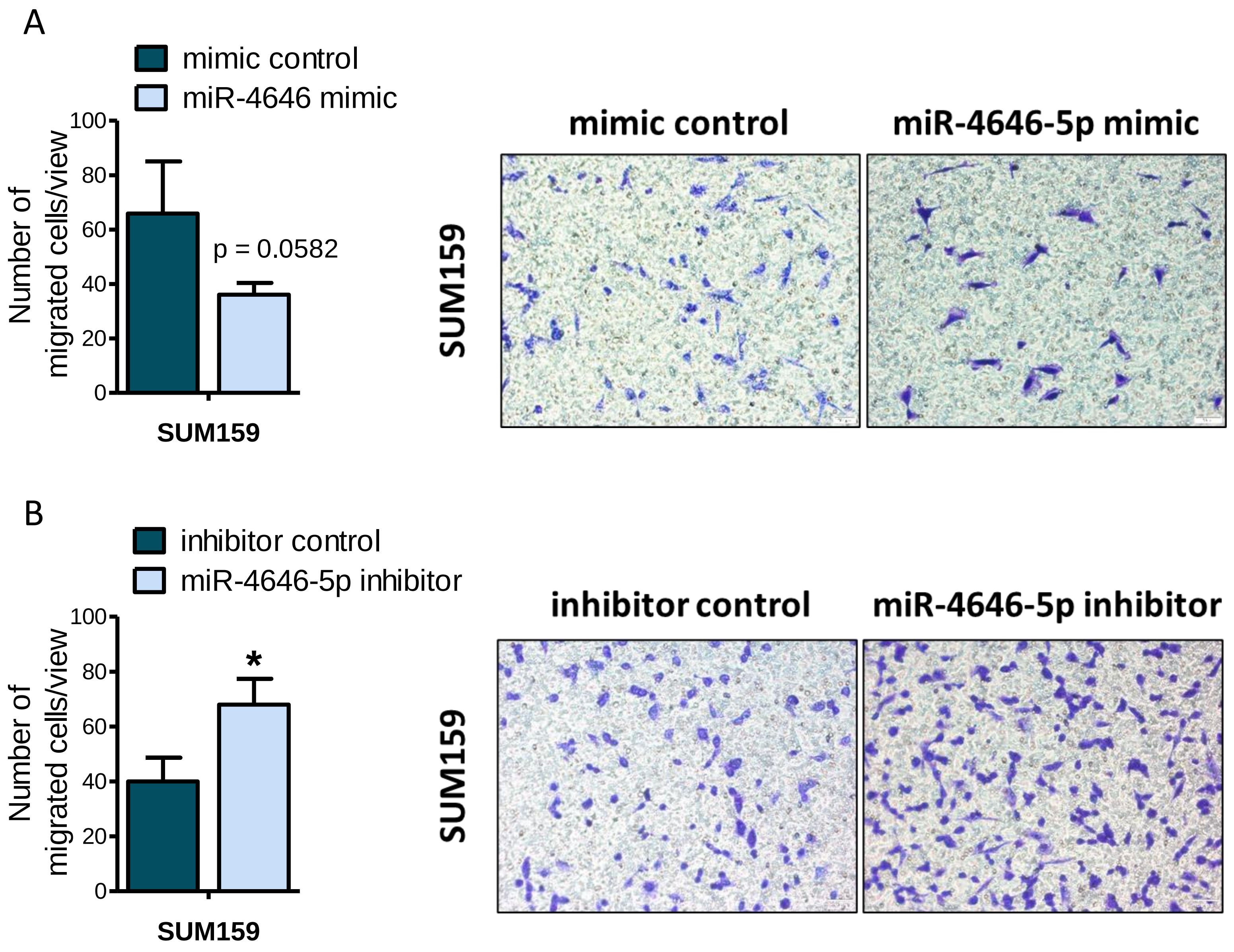

Supplementary Figure S5. The effect of (A) transient miR-4646-5p mimic, and (B) transient inhibitor transfection on the migration of SUM159 cells was investigated in transwell assays. In each transwell, cells were counted in five representative fields of view with 40× magnification (representative images of 20× magnification are shown on the right) and are presented as mean ± SD (n = 3; * $p \leq 0.05$).

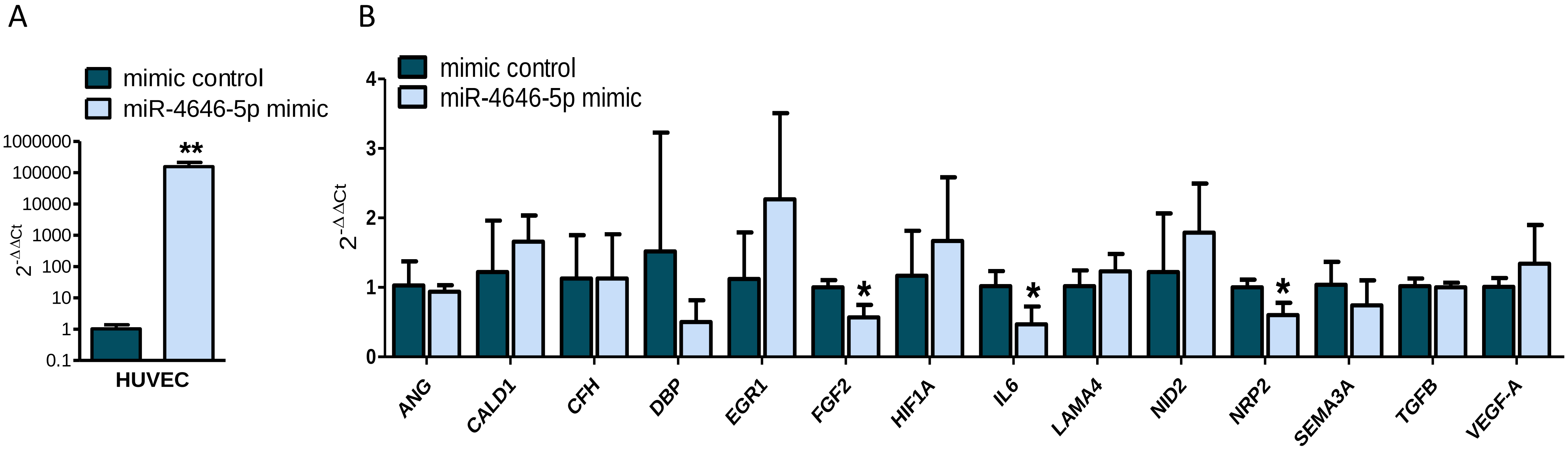

Supplementary Figure S6. Expression of angiogenesis-related genes in human umbilical vein endothelial cells (HUVECs) upon miR-4646-5p mimic transfection. (A) Transient overexpression of miR-4646-5p by mimic transfection (48 h) in human umbilical vein endothelial cells (HUVECs) measured by RT-qPCR, depicted relative to the control (mean ± SD; n = 3; $^{**}p \leq 0.01$). **(B)** Expression of angiogenesis-related genes was measured by RT-qPCR 48 h after miR-4646-5p mimic or control transfection (n = 3; mean ± SD; $^{*}p \leq 0.05$). ANG…angiogenin, CALD1…caldesmon, CFH…complement factor H, DBP…D-box binding PAR BZIP transcription factor, EGR1…early growth response 1, FGF2…fibroblast growth factor 2, HIF1A…hypoxia-inducible factor 1 subunit alpha, IL-6…interleukin 6, LAMA4…laminin subunit alpha 4, NID2…nidogen 2, NRP2…neuropilin 2, SEMA3A…semaphorin 3A, TGFB…transforming growth factor beta, VEGF-A…vascular endothelial growth factor A.

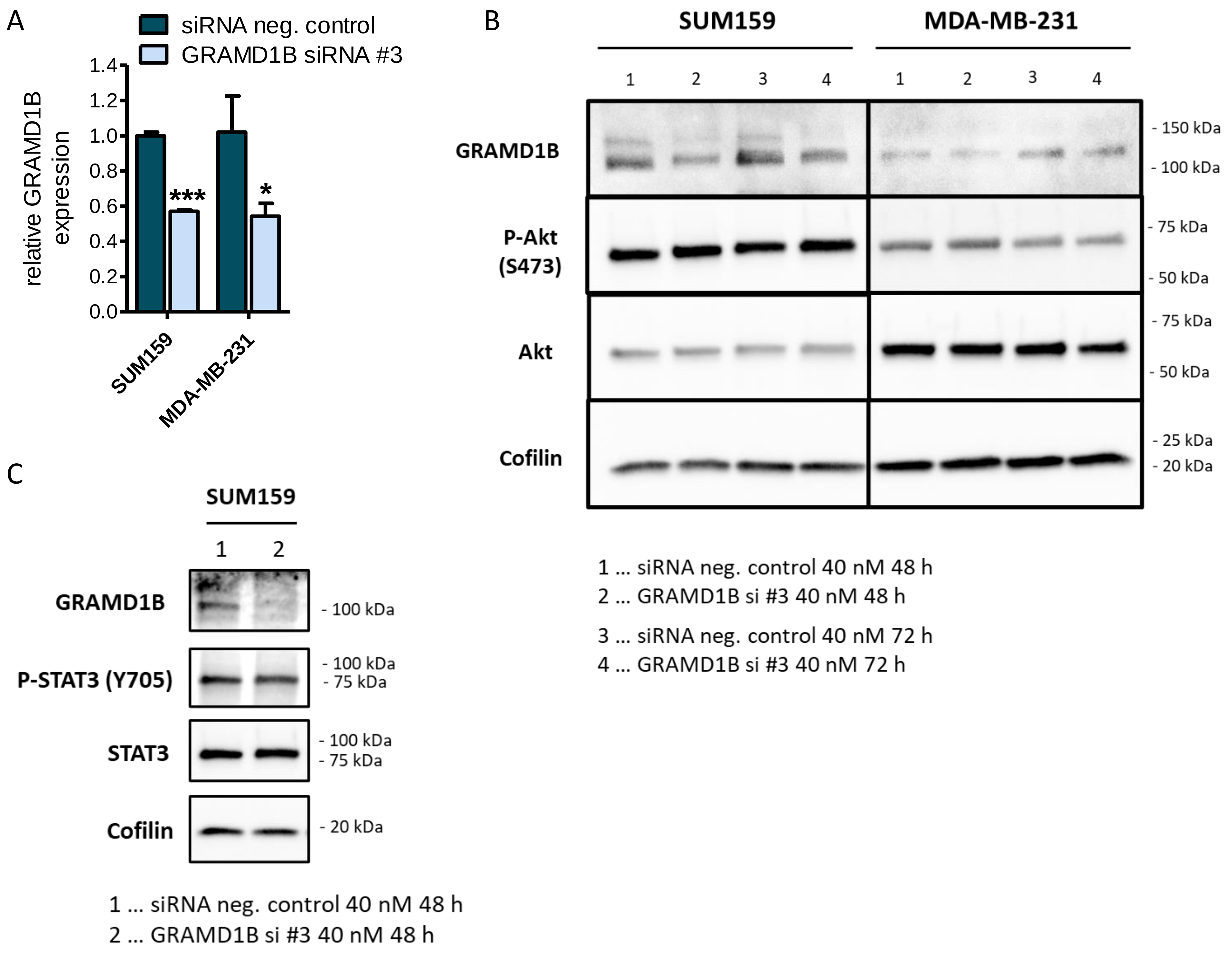

Supplementary Figure S7. SiRNA-mediated knockdown of the GRAM Domain-Containing Protein 1B (GRAMD1B) in two TNBC cell lines and the impact on AKT and STAT3 phosphorylation. **(A)** GRAMD1B knockdown-efficiency on the RNA level as determined by RT-qPCR 48 h after transduction (mean ± SD; n = 3; *p ≤ 0.05, ***p ≤ 0.001). **(B)** GRAMD1B knockdown on the protein level 48 h and 72 h after transduction as determined by Western blotting. In addition, the impact on AKT phosphorylation was investigated. **(C)** The impact of GRAMD1B knockdown on STAT3 phosphorylation in SUM159 cells is shown in a representative Western Blot.

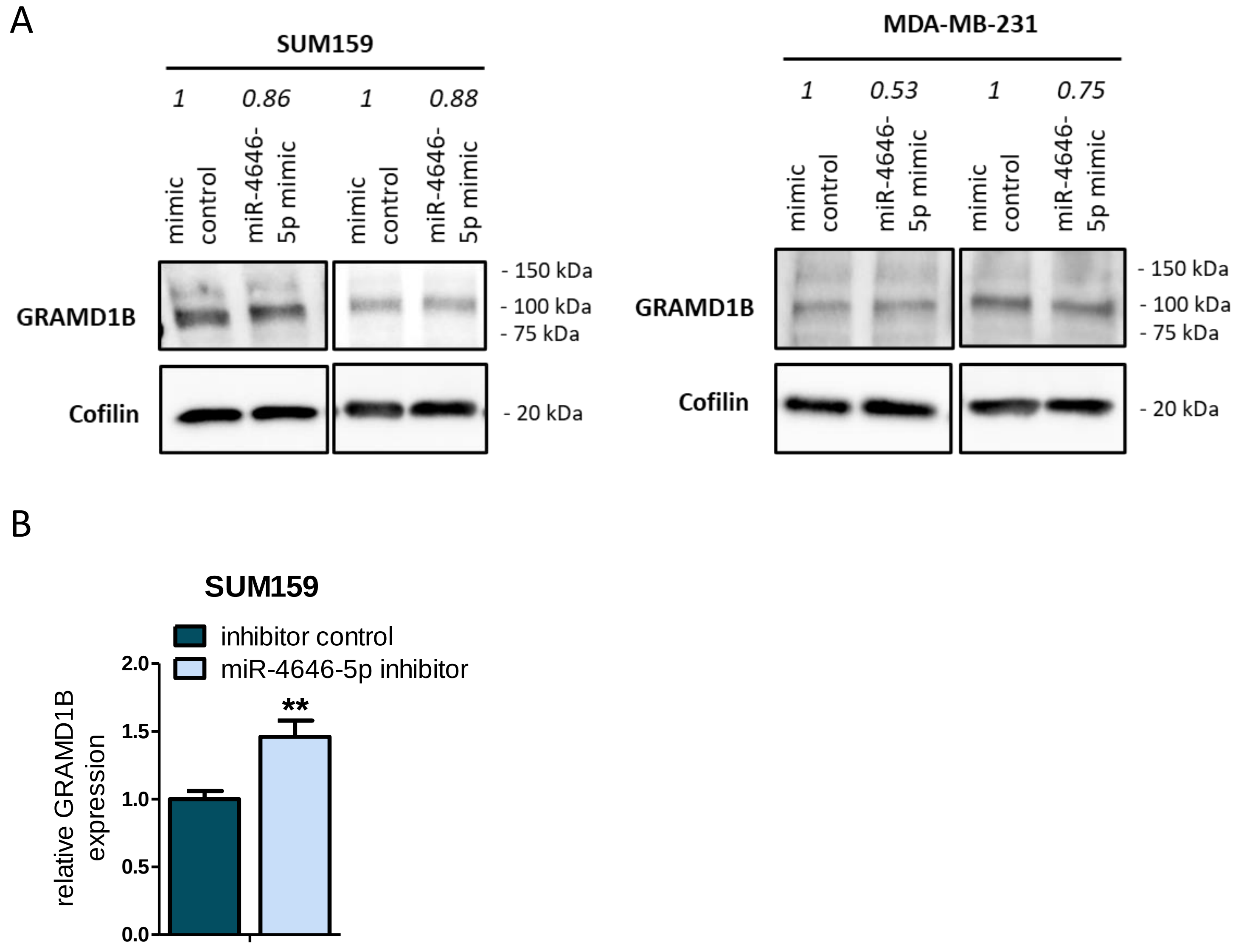

Supplementary Figure S8. Effect of miR-4646-5p on GRAMD1B protein and expression. (A) Impact of 48 h miR-4646-5p mimic transfection on GRAMD1B protein in SUM159 and MDA-MB-231 cells as determined by Western Blotting. Fold changes in signal intensity normalized to Cofilin and relative to the mimic controls are indicated. **(B)** Impact of 48 h miR-4646-5p inhibitor transfection on GRAMD1B expression in SUM159 cells as determined by RT-qPCR (mean ± SD; n = 3; $^{**}p \le 0.01$).

Supplementary Table S1. Sequences of Primers

Gene	Primer orientation	Sequence (5'->3')
BAK	Forward	TGC CCT CTG CTT CTG AGG A
	Reverse	TTC CTG CTG ATG GCG GTA AA
BAX	Forward	AGG GTG GTT GGG GGC TG
	Reverse	AAA GTA GGA GAG GAG GCC GT
PUMA	Forward	TGA AAT TTG GCA TGG GGT CTG
	Reverse	CTC CCT GGG GCC ACA AAT
BAD	Forward	TGT GGA CTC CTT TAA GAA GGG AC
	Reverse	CAC CAG GAC TGG AAG ACT CG
NOXA	Forward	GGA GAT GCC TGG GAA GAA GG
	Reverse	CAC TCG ACT TCC AGC TCT GC
FGF2	Forward	GCTGTACTGCAAAAACGGGG
	Reverse	TAGCTTGATGTGAGGGTCGC
IL6	Forward	ACCCCCAATAAATATAGGACTGGA
	Reverse	GAGAAGGCAACTGGACCGAA
NRP2	Forward	GTACCAGATTGTGTTCGAGGG
	Reverse	TTCTCACCTGCAAAAGCCGA
GCSF	Forward	TTGAGCCAACTCCATAGCGG
	Reverse	TCCCAGTTCTTCCATCTGCTG
GRAMD1B	Forward	TCAAACATGTGGCAGGTTCC
	Reverse	TAAGGATGACCAGCAGCACC
GAPDH	Forward	AAGGTCGGAGTCAACGGATTT
	Reverse	ACCAGAGTTAAAAGCAGCCCTG
U6	Forward	CTCGCTTCGGCAGCACA
	Reverse	AACGCTTCACGAATTTGCGT

Supplementary Table S2. Top 20 downregulated genes (ranked by fold change) after miR-4646-5p mimic transfection in SUM159 as determined by RNA-seq.

Ensembl Gene ID	Gene Name	Log2 fold change	Adjusted p-value
**ENSG00000108342**	*CSF3*	-1.99	5.285e-12
**ENSG00000136244**	*IL6*	-1.79	7.017e-26
**ENSG00000115009**	*CCL20*	-1.65	0.000274
**ENSG00000163568**	*AIM2*	-1.54	0.001698
**ENSG00000023171**	*GRAMD1B*	-1.43	1.808e-12
**ENSG00000269927**	*AC004817.3*	-1.39	0.008273
**ENSG00000163739**	*CXCL1*	-1.32	2.192e-05
**ENSG00000273760**	*AC245041.1*	-1.31	5.391e-05

**ENSG00000188766**	*SPRED3*	-1.28	0.0009108
**ENSG00000236453**	*AC003092.1*	-1.27	0.0001296
**ENSG00000278266**	*AC079949.2*	-1.13	0.0408863
**ENSG00000196611**	*MMP1*	-1.12	2.307e-13
**ENSG00000125538**	*IL1B*	-1.09	0.0303237
**ENSG00000130021**	*PUDP*	-1.08	4.005e-19
**ENSG00000164400**	*CSF2*	-1.07	4.520e-06
**ENSG00000197249**	*SERPINA1*	-1.07	0.0243591
**ENSG00000170345**	*FOS*	-1.06	1.498e-06
**ENSG00000120738**	*EGR1*	-1.06	4.520e-06
**ENSG00000232774**	*AL355916.1*	-1.06	6.163e-07
**ENSG00000280219**	*AC093908.1*	-1.06	0.0368737

Supplementary Table S3. Target binding sites for miR-4646-5p and its target GRAM domain-containing protein 1B (GRAMD1B) as predicted by the TargetScan algorithm.

<table>
<tr><th>Binding site number</th><th>Position</th><th>Predicted consequential pairing of target region (top) and miR-4646-5p (bottom)</th><th>Context++ score percentile</th></tr>
<tr><td>1</td><td>Position 1623-1629 of GRAMD1B 3' UTR</td><td>5' ...AAGAAACCCCAGAUGUCCCAGAU...
||||||
3' AGGGAGUCGAGGAGAAGGGUCA</td><td>84</td></tr>
<tr><td>2</td><td>Position 1632-1638 of GRAMD1B 3' UTR</td><td>5' ...CAGAUGUCCCAGAUGUCCCAGAA...
||||||
3' AGGGAGUCGAGGAGAAGGGUCA</td><td>86</td></tr>
<tr><td>3</td><td>Position 2097-2104 of GRAMD1B 3' UTR</td><td>5' ...CAGAAGCCAAAACUAUUCCCAGA...
|||||||
3' AGGGAGUCGAGGAGAAGGGUCA</td><td>97</td></tr>
<tr><td>4</td><td>Position 2299-2305 of GRAMD1B 3' UTR</td><td>5' ...UACCUCUCAGGACCA-UUCCCAGG...
||||| |||||||
3' AGGGAGUCGAGGAGAAGGGUCA</td><td>33</td></tr>
<tr><td>5</td><td>Position 3234-3240 of GRAMD1B 3' UTR</td><td>5' ...CCCAGAGGCUGAAGCUUCCCAGU...
||| |||||||
3' AGGGAGUCGAGGAG-AAGGGUCA</td><td>33</td></tr>
<tr><td>6</td><td>Position 3272-3278 of GRAMD1B 3' UTR</td><td>5' ...AGGGCAGUGUCUGCAUUCCCAGG...
|||||||
3' AGGGAGUCGAGGAGAAGGGUCA</td><td>33</td></tr>
<tr><td>7</td><td>Position 3878-3884 of GRAMD1B 3' UTR</td><td>5' ...UCUGGAUGCUUGUGGUCCCAGAA...
||| ||||||
3' AGGGAGUCGAGGAGAAGGGUCA</td><td>78</td></tr>
</table>

Supplementary Table S4. Gene Ontology (GO) enrichment analysis of genes that were significantly downregulated by miR-4646-5p in an RNA-seq analysis. The table presents significantly enriched GO biological processes, the total number of genes annotated to these processes, how many of these genes were downregulated by miR-4646-5p (termed enriched gene number), the number of genes that would have been expected to be deregulated randomly, the fold enrichment, whether the enrichment is positive or negative (+/-), the raw p-values and the false discovery rate (FDR)-corrected p-values.

GO molecular function	Total gene number	Enriched gene number	Expected gene number	Fold enrichment	+/-	raw p-value	FDR-corrected p-value
**transmembrane receptor protein tyrosine kinase activator activity**	14	4	0.21	18.67	+	1.31E-04	4.34E-02
**molecular function regulator activity**	2031	53	31.07	1.71	+	1.35E-04	4.20E-02
**epidermal growth factor receptor binding**	33	6	0.50	11.88	+	2.40E-05	1.08E-02
**growth factor receptor binding**	141	13	2.16	6.03	+	6.30E-07	6.27E-04
**protein binding**	14448	271	221.05	1.23	+	8.81E-11	4.39E-07
**binding**	16594	291	253.88	1.15	+	8.56E-09	2.13E-05
**protein serine kinase activity**	364	19	5.57	3.41	+	6.31E-06	3.14E-03
**protein kinase activity**	583	26	8.92	2.91	+	2.06E-06	1.47E-03
**kinase activity**	765	28	11.70	2.39	+	3.35E-05	1.39E-02
**transferase activity, transferring phosphorus-containing groups**	919	31	14.06	2.20	+	5.42E-05	2.07E-02
**phosphotransferase activity, alcohol group as acceptor**	692	28	10.59	2.64	+	4.96E-06	3.08E-03
**cytokine receptor binding**	272	14	4.16	3.36	+	1.21E-04	4.30E-02
**protein serine/threonine kinase activity**	433	22	6.62	3.32	+	1.77E-06	1.47E-03
**enzyme binding**	2103	59	32.17	1.83	+	6.02E-06	3.33E-03
